# Integrative physiological, biochemical, and proteomic analysis of the leaves of two cotton genotypes under heat stress

**DOI:** 10.1371/journal.pone.0316630

**Published:** 2025-01-09

**Authors:** Asia Perveen, Sheheryar Sheheryar, Fiaz Ahmad, Ghazala Mustafa, Arlindo Alencar Moura, Francisco A. P. Campos, Gilberto B. Domont, Umar Nishan, Riaz Ullah, Mohamed A. Ibrahim, Fábio C. S. Nogueira, Mohibullah Shah

**Affiliations:** 1 Department of Biochemistry, Bahauddin Zakariya University, Multan, Pakistan; 2 Department of Biochemistry and Molecular Biology, Federal University of Ceara, Fortaleza, Brazil; 3 Department of Animal Science, Federal University of Ceara, Fortaleza, Brazil; 4 Physiology/Chemistry Section, Central Cotton Research Institute, Multan, Pakistan; 5 Faculty of Biological Sciences, Department of Plant Sciences, Quaid-i-Azam University, Islamabad, Pakistan; 6 Department of Biochemistry, Proteomic Unit, Institute of Chemistry, Federal University of Rio de Janeiro, Rio de Janeiro, Brazil; 7 Hainan International Joint Research Center of Marine Advanced Photoelectric Functional Materials, College of Chemistry and Chemical Engineering, Hainan Normal University, Haikou, PR China; 8 Department of Chemistry, Kohat University of Science & Technology, Kohat, Pakistan; 9 Department of Pharmacognosy, College of Pharmacy, King Saud University, Riyadh, Saudi Arabia; 10 Department of Pharmaceutics, College of Pharmacy, King Saud University, Riyadh, Saudi Arabia; University of Agriculture Faisalabad, PAKISTAN

## Abstract

Cotton (*Gossypium hirsutum* L.), a crucial global fibre and oil seed crop faces diverse biotic and abiotic stresses. Among these, temperature stress strongly influences its growth, prompting adaptive physiological, biochemical, and molecular changes. In this study, we explored the proteomic changes underscoring the heat stress tolerance in the leaves of two locally developed cotton genotypes, i.e., heat tolerant (GH-Hamaliya H_tol_) and heat susceptible (CIM-789 H_sus_), guided by morpho-physiological and biochemical analysis. These genotypes were sown at two different temperatures, control (35°C) and stress (45°C), in a glasshouse, in a randomized complete block design (RCBD) in three replications. At the flowering stage, a label-free quantitative shotgun proteomics of cotton leaves revealed the differential expression of 701 and 1270 proteins in the tolerant and susceptible genotypes compared to the control, respectively. Physiological and biochemical analysis showed that the heat-tolerant genotype responded uniquely to stress by maintaining the net photosynthetic rate (*Pn*) (25.2–17.5 μmolCO_2_m^-2^S^-1^), chlorophyll (8.5–7.8mg/g FW), and proline contents (4.9–7.4 μmole/g) compared to control, supported by the upregulation of many proteins involved in several pathways, including photosynthesis, oxidoreductase activity, response to stresses, translation, transporter activities, as well as protein and carbohydrate metabolic processes. In contrast, the distinctive pattern of protein downregulation involved in stress response, oxidoreductase activity, and carbohydrate metabolism was observed in susceptible plants. To the best of our knowledge, this is the first proteomic study on cotton leaves that has identified more than 8000 proteins with an array of differentially expressed proteins responsive to the heat treatment that could serve as potential markers in the breeding programs after further experimentation.

## Introduction

*Gossypium hirsutum* L. (Cotton) is a prominent crop that covers around 2.5% of the world’s cultivated areas. It is also known as a dual-purpose crop, utilized for both its natural fiber and its 4% contribution to global vegetable oil production. Cotton is one of the finest sources of plant protein and the 5^th^ most important oilseed crop after soybean, sunflower, palm, and canola [1].

Pakistan is especially vulnerable to climate change, and the average temperature has risen by 0.5°C from the previous 30 years [2]. Due to these climatic changes, cotton crops face different abiotic stresses like drought [3], salinity [4]and extreme temperature stress [5] which have a deleterious effect at all developmental stages, resulting in a remarkable reduction of yield by about 73% and fibre quality [6]. The cotton plant is more susceptible to heat stress during the reproductive phase, which can cause delayed flowering [7], flower sterility, and boll shedding problems [5]. While, during the vegetative phase, heat stress affects plant physiology by reducing the number of fruiting branches, number of nodes on the main stem and shedding 50% of premature bolls, which negatively impacts crop yield [8].

Current climatic conditions can have profound effects on plants at physiological, cellular, molecular, and enzymatic levels. High day temperatures lead to higher transpiration, which lowers water potential and thereby disrupts several physiological processes, similar to drought stress [9]. In many genotypes, heat stress can inhibit stomatal conductance and the net photosynthetic rate [10]. Moreover, heat stress causes changes at the subcellular level, inhibiting photosynthesis by changing the thylakoid structures [11,12]. Heat stress tolerance can be regulated by modifications in cell membraneʼs protein composition, tissue water content, lipid activity, and primary and secondary metabolites [13]. At the molecular level, plants respond to stress by altering gene expression, which results in various changes in protein synthesis (up or downregulation) and subsequently influences their biological activities [14]. These proteins participate in regulating physiological and biochemical processes to maintain cellular homeostasis under abiotic stresses. Under heat stress, protein concentrations might undergo drastic changes, elucidating the plant’s coping mechanisms.

Previously, proteomics approaches have been utilized to study the effects of abiotic stresses by identifying the differentially expressed proteins and the role of these proteins in diverse biological pathways that respond to these drastic conditions [15]. Different studies on the cotton genotypes have been conducted under abiotic stresses such as drought stress to see the morpho-physiological and proteomic responses [16]. However, little attention has been given to the proteomic investigation of the effect of high-temperature stress on cotton plants. Heat stress, in particular, disrupts photosynthesis and protein functions, making it a critical factor in cotton’s development. Biotic stresses, such as pests and pathogens, further complicate plant health by damaging tissues and spreading diseases [17]. This study primarily focuses on heat stress, examining cotton’s physiological, biochemical, and proteomic changes to better understand and develop heat-tolerant genotypes.

To broaden the genetic diversity of different genotypes, traditional breeding methods are being utilized, which aids in producing high-performing genotypes with desired traits [18]. For this study, selecting locally developed tolerant genotypes (GH-Hamaliya) that maintain the total number of bolls per plant, minimizing flower and boll shedding to enhance cotton production under heat stress. Comparing this to the susceptible genotype (CIM-789) lacking such adaptations is necessary to identify the proteins participating in the heat stress tolerance mechanism. The general goal of this study is to enhance our understanding of heat stress tolerance mechanisms in cotton utilizing an integrative approach while specifically identifying differentially expressed proteins and elucidating their roles in stress response.

## Material and methods

**Fig 1:**
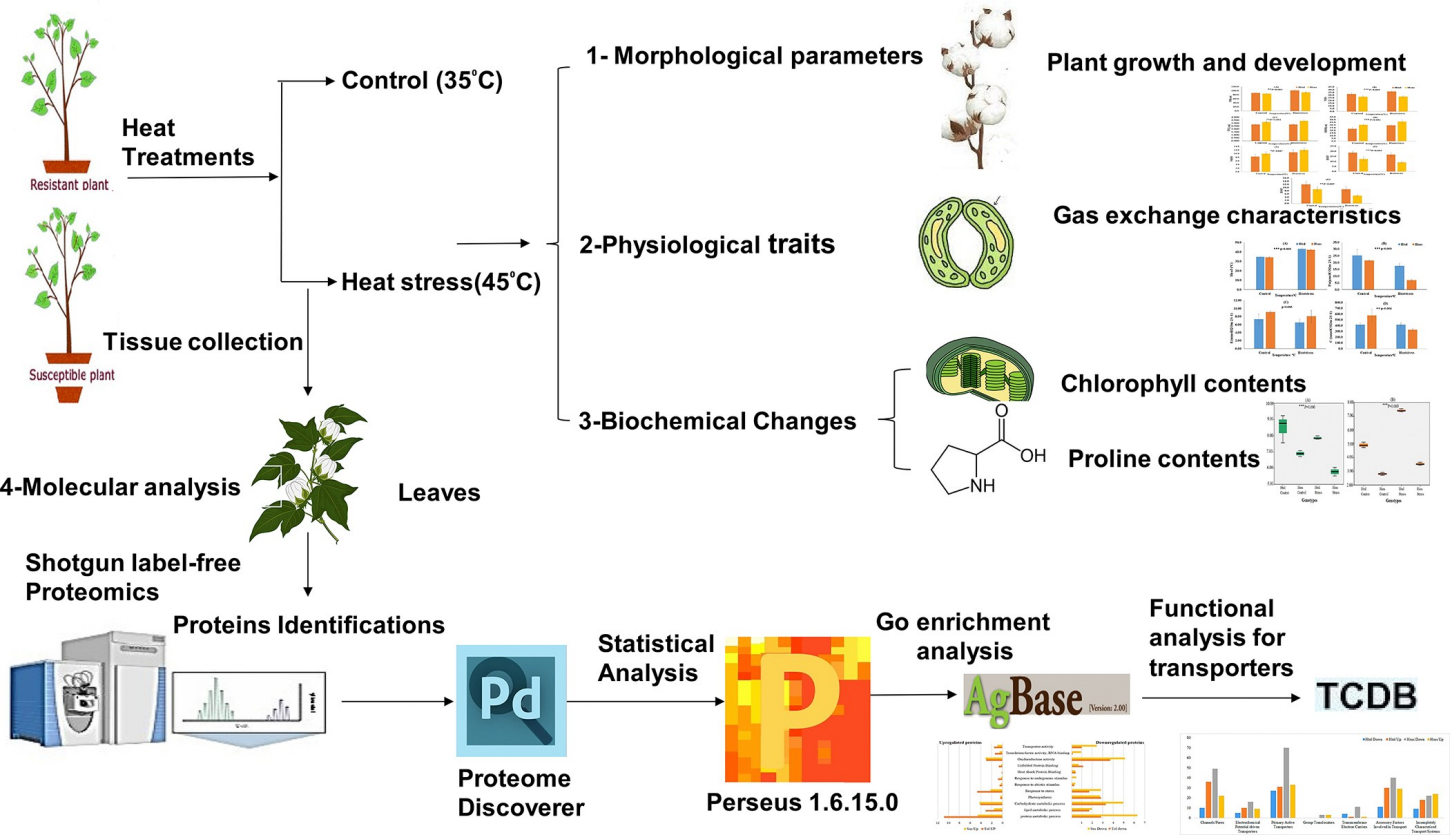
Schematic diagram of the experimental design. Plants were treated at 35°C and 45°C and subjected to morphological, physiological, biochemical, and shotgun proteomic analysis.

A detailed visual representation of the experimental design is shown in Fig 1. Cotton genotypes were grown at two different temperatures and subjected to morphological, physiological, biochemical, and proteomic analysis as explained below.

### Plants treatment

*Gossypium hirsutum* L. seeds of two cotton genotypes, heat-tolerant (GH-Hamaliya) (H_tol_) and heat-susceptible (CIM-789) (H_sus)_, were collected from the Central Cotton Research Institute (CCRI) Multan in April 2021. Both genotypes were locally developed and screened during continuous annual field experiments for heat stress tolerance based on the differences in their morphological characters, fruit positioning parameters, plant structure and development, leaf shapes, cell membrane thermostability (CMT%), boll weight, and yield parameters. These morphological and physiological variations led to the selection of both genotypes for further analysis to comprehend the mechanism of heat stress tolerance through differently expressed proteins (DEPs) under heat stress. The two cotton varieties were cultivated in clay pots (18.5 cm height × 22 cm width) filled with soil (silt loam) at optimal temperatures (35°C/25°C; day/night) and above-normal (heat stress) temperatures (45°C/32°C; day/night) in the greenhouse of the Central Cotton Research Institute (CCRI) in Multan. The soil composition was determined in the physiology lab of Central Cotton Research Institute Multan. Soil samples were collected, and physio-chemical analyses of the soil revealed that the soil is silt loam in texture and alkaline in reaction. Soil pH was 8.15, electrical conductivity was 2.85 mS cm-1, organic matter content was 0.71%, extractable phosphorus level was 21.4 mg kg-1 soil, and extractable potassium was 153.8 mg kg-1. Three cottonseeds of both varieties were planted in each pot and left to germinate at varying temperatures. Transpiration rate and soil humidity were measured [19] and plants were properly watered and fertilized with full-strength Hoagland nutrient solution and recommended fertilizers [20]. The experiment was laid out in a randomized complete block design (RCBD) [21] with three biological replicates. For each genotype, three pots and 5 plants were used in each replication for data analysis. Plants were developed until the boll formation stage (120–150 days after planting). Ten leaves were collected from each plant at the flowering stage (90 DAP) and examined for morphological, physiological, biochemical, and proteomic analysis.

### Plant structure and development analysis

Five plants of heat-tolerant and susceptible varieties with no mechanical issues from three biological replications were selected for morphological and physiological parameter assessment. According to [22] earliness parameters, including main stem height, number of nodes on the main stem, inter-nodal distance, sympodial node number bearing the first effective boll, sympodial node height bearing the first effective boll, first square appearance day, and first flower appearance day, were recorded.

### Gas exchange characteristics

Using CI-340 portable photosynthesis equipment at 90 DAP, photosynthesis and other gas exchange parameters were recorded simultaneously from the third completely grown leaf in two temperature regimes. The measurements were taken in a measuring chamber with a temperature that corresponds to the daytime growth temperatures, i.e., ±35°C, and with a light intensity of approximately 1500 μmol m^−2^ s^−1^. The leaf chamber [CO_2_] was set to up to 750 ppm, and the relative humidity was adjusted to the ambient level (around 50%). The flow rate through the chamber was set to 350 mol s^−1^. Net photosynthesis (*P*_*n*_) and the fluorescence in light (Fv′/Fm′) were calculated as the total coefficient of variation (CV%) until a value below 0.5% was reached. Transpiration rate (*E*), net photosynthetic rate, and stomatal conductance were measured with the photosynthesis equipment with consideration of leaf area and incoming and outgoing flow rates, whereas water use efficiency (WUE) was anticipated as the ratio of *Pn*to *E*.

### Total chlorophyll analysis

The contents of leaf photosynthetic pigments (chlorophyll a and b) were measured as reported earlier [23]. One set of leaf samples was taken at 90 DAP from the third highest fully expanded leaves in each genotype and temperature treatment. A 0.1 g of fresh leaf material was homogenized with a 20-ml mixture of acetone, pure ethanol, and distilled water (in a 4.5:4.5:1 ratio). The tubes were kept in darkness until the color transitioned to white, then centrifuged at 5000 rpm for 5 minutes. The resulting supernatant was adjusted to a volume of 50 ml using a volumetric flask. The solution extracted has its absorbance (O.D.) measured at wavelengths of 663 and 645 nm (indicative of chlorophyll a and b). The concentrations of chlorophyll (a and b) pigments were determined following [24] and expressed as per leaf area (μg cm^−2^). Total chlorophyll content was calculated by adding up the values of chlorophyll a and b.

### Determination of proline content

Fully expanded leaves from cotton plants were sampled and processed for proline content determination as per previous procedures [25]. To prepare acid ninhydrin, 1.25 g of ninhydrin was heated to complete dissolution in a solution of glacial acetic acid (30 ml) and 6 M phosphoric acid (20 ml) while being continuously stirred. This solution was kept at 4°C for its stability for 24 hours. Almost 0.5–1.0 g of leaf tissues were ground with 10 ml of sulfosalicylic acid (3%) and then filtered through the Whatman No. 2 filter paper. Following filtration, 2 ml of the filtrate was reacted in a test tube with 2 ml of acid ninhydrin and 2 ml of glacial acetic acid. The test tube was moved to an ice bath to end the reaction, which predictively lasted for an hour at a temperature of 100°C. The reaction product mixture was then extracted with the addition of 4 ml of toluene and vigorously shaken for 15 to 20 seconds. The chromophore-containing toluene was taken out of the aqueous phase and heated to room temperature. The absorbance was measured at 520 nm while using toluene as a baseline. The following formula was used to calculate the proline concentration using a standard curve and fresh weights:

[(μg proline/ml toluene)/115.5 μg/μmole] / [(g sample)/5] = μ moles proline/g leaf fresh weight

### Statistical analysis of morphological, physiological, and biochemical parameters

Statistical analysis was performed to evaluate the effect of heat stress on H_tol_ and H_sus_ genotypes under control and stress conditions by using the SPSS IBM 20 tool and applying one-way ANOVA separately in each case, i.e., tolerant control vs tolerant stress and susceptible control vs susceptible stress for each dependent variable.

Further, to investigate the heat tolerance mechanism in cotton leaves, the two varieties were subjected to a label-free quantitative shotgun proteomics analysis.

### Protein extraction

Leaf samples from both genotypes were freeze-dried, ground into fine powder in the presence of liquid nitrogen using a mortar and pestle, and stored at -20°C for further use [26]. Protein extraction was performed using a previously established protocol [27]. Briefly, 2.0 g of fine ground powder was mixed with 10% PVPP of the sample weight in a 50-ml falcon tube. Then a precooled 10% TCA/acetone solution was added to fill the tube. After thorough homogenization, the mixture was centrifuged at 12,000 rpm at 4°C for 5 minutes. The supernatant was properly taken out by using a pipette. To get rid of any remnant of TCA, the pellet was washed with 80% methanol mixed with 0.1 M ammonium acetate (pH above 7), followed by centrifugation at 12,000 rpm at 4°C for 5 minutes. The supernatant was discarded, and the pellets were washed with the cooled acetone solution. After drying and removing the acetone residues from the pellets, 1.5 ml of extraction buffer, i.e., 1:1 phenol (pH 7.9) 750 μl and 750 μl of SDS buffer (30% sucrose, 2% SDS, 0.1 M Tris-HCl, pH 8.0, 5% β-mercaptoethanol, and 1 mM PMSF) were added, well mixed, and placed on a shaker incubator for 5 minutes at 4°C, followed by centrifugation at 12,000 ×g for 10 minutes at 4°C. After collecting the top layer of phenol, 4–8 volumes of methanol containing 0.1 M ammonium acetate were added, and the mixture was allowed to precipitate overnight at -30°C. The phenolic supernatant was removed by centrifugation, and the pellet was cleaned three times with pre-cooled methanol and acetone, respectively. The pellet was dried and solubilized by adding urea (7M) and thiourea (2M) and stored at -20°C for further analysis [28]. Protein quantification was carried out by using the Bradford assay [29].

### Protein digestion and sample preparation for nano-LC-MS/MS

For in-solution digestion of extracted proteins, 100 μg of protein sample was utilized with some modifications of the method described earlier [30,31]. A 100-μg protein aliquot was taken, reduced with 10 mM DTT for 1 hour at 30°C, alkylated with 40 mM iodoacetamide (IAA) for 30 minutes at room temperature, and then covered to protect from light. The urea concentration was diluted by adding 50 mM ammonium bicarbonate in a 1:9 (sample: ammonium bicarbonate). Trypsin (Promega) was then used to digest the proteins for 18 hours at 37°C. Trifluoroacetic acid (TFA) (10%) was added to the mixture to stop the digestion, and the peptides were passed through a C18 Poros R2 resin (Applied Biosystems) column for cleaning. The column was washed three times with 100% acetonitrile, followed by the application of the peptides. After passing, the peptides were cleaned of any salt and detergent residue using 200 μl of 0.1% TFA. The peptides were eluted in acetonitrile with 0.1% TFA, 50% ACN, and 50 μl of 0.1% TFA and 70% ACN. A Thermo Fisher SRF 110-speed vacuum concentrator was used to concentrate the eluted peptides. The peptides were solubilized in 15 μl of 0.1% formic acid to ensure peptide recovery.

### Nano-LC-MS/MS analysis

Aliquots containing 6 μg of peptides from every sample were diluted with 0.1% FA to make a final concentration of 0.5 μg/μL. The samples were then analyzed in a Thermo Fisher Scientific nanoLC-EASY 1000 system connected online to an ESI-Q Exactive Plus Orbitrap mass spectrometer. A 4 μL sample was eluted from a C18 ReproSil-Pur (C18-AQ) trap column (Maisch GmbH in Ammerbuch, Germany). It was separated into a C18 column (20 cm length × 75 μm diameter) with a 3 μm particle diameter. The chromatographic separation was taken for 120 minutes with a separation flow rate of 200 nL/min, followed by a gradient of 5% to 30% of phase A (H_2_O 95%, ACN 5%, FA 0.1%) for 100 minutes and 30% to 45% of phase B (H_2_O 5%, ACN 95%, FA 0.1%) for 9 minutes, ending on isocratic 95% of phase B for 5 minutes. The peptides were positively polarized and ionized at 2.30 kV in an ESI source. Data was collected in DDA mode while the ion transfer capillary was maintained at 200°C. The MS1 spectra comprised a complete scan in the m/z range of 350.0–1800.0, including a minimum intensity of 2000 and a resolution of 60,000 FWHM (for m/z 400). The 20 most intense precursor ions were fragmented by HCD for MS2 acquisition with a resolution of 7,500 FWHM (for m/z 400), normalized CE (collision energy) of 30 V, an isolation window of 2.5 m/z, and dynamic exclusion of 45 s.The mass spectrometry data have been deposited to the ProteomeXchange Consortium [32] via the PRIDE [33] partner repository with the dataset identifier PXD056003.

### Database search

The Xcalibur v2.1 software program was used to view the MS/MS raw file of each sample of both genotypes. The program Xcalibur v.2.1 (Thermo Scientific) was used to analyze the raw spectral data for determining the signal intensity between technical and biological replicates. The search database was created by downloading and merging the *G*. *hirsutum* L. nuclear-encoded proteins from Uniprot with those of the plastids and mitochondrial-encoded proteins downloaded from the NCBI database. The spectral correspondence of tryptic peptides was used to identify the proteins. The Proteome Discoverer 2.5 v software (Thermo Scientific) with the SEQUEST algorithm was employed to search for spectra for protein identification, and PSMs (Peptide Spectrum Match) were obtained [34]. An FDR of 1% and 5% were used at the peptide and protein levels, respectively. The gene ontology annotation was used to functionally classify the differentially expressed proteins using the AgBase tool and database (https://agbase.arizona.edu/index.html). Similarly, the proteins were also annotated using the TCDB databases (https://www.tcdb.org/) classification system for transport-related proteins [35].

### Statistical analysis

Statistical analysis of the data was conducted using Perseus software (1.6.14). Proteins discovered in a sample with at least one unique peptide and found in at least one technical replicate of two biological replicates were deemed identified and included in the subsequent analysis. Data normalization was performed through log2(x) transformation, followed by histogram analysis to assess the distribution of transformed data. Differential protein expression was evaluated using a two-sample Student’s t-test with a significance threshold of p ≤ 0.05, where significance was determined by -log10(p-value). Principal component analysis (PCA) was employed to explore variance in the data, applying the Benjamini-Hochberg method to control the false discovery rate (FDR) at 0.05. A volcano plot was generated to visualize differential expression, utilizing a t-test with 250 permutations and an FDR of 0.05. Proteins identified as significant by the t-test were further analyzed through z-score normalization, followed by Pearson correlation analysis to evaluate the relationships between the normalized protein expressions. Hierarchical clustering was then performed using Euclidean distance as the metric, average linkage for clustering, and the number of clusters set to 300.

## Results

For evolving heat-tolerant varieties, it is necessary to understand the morphological, physiological, and biochemical changes taking place in tolerant and susceptible genotypes under heat stress conditions. Two cotton genotypes, H_tol_ and H_sus_,were used to evaluate their performance at a normal temperature of 35°C (control) and a high temperature of 45°C (heat stress).

### Morphological measurement

**Fig 2:**
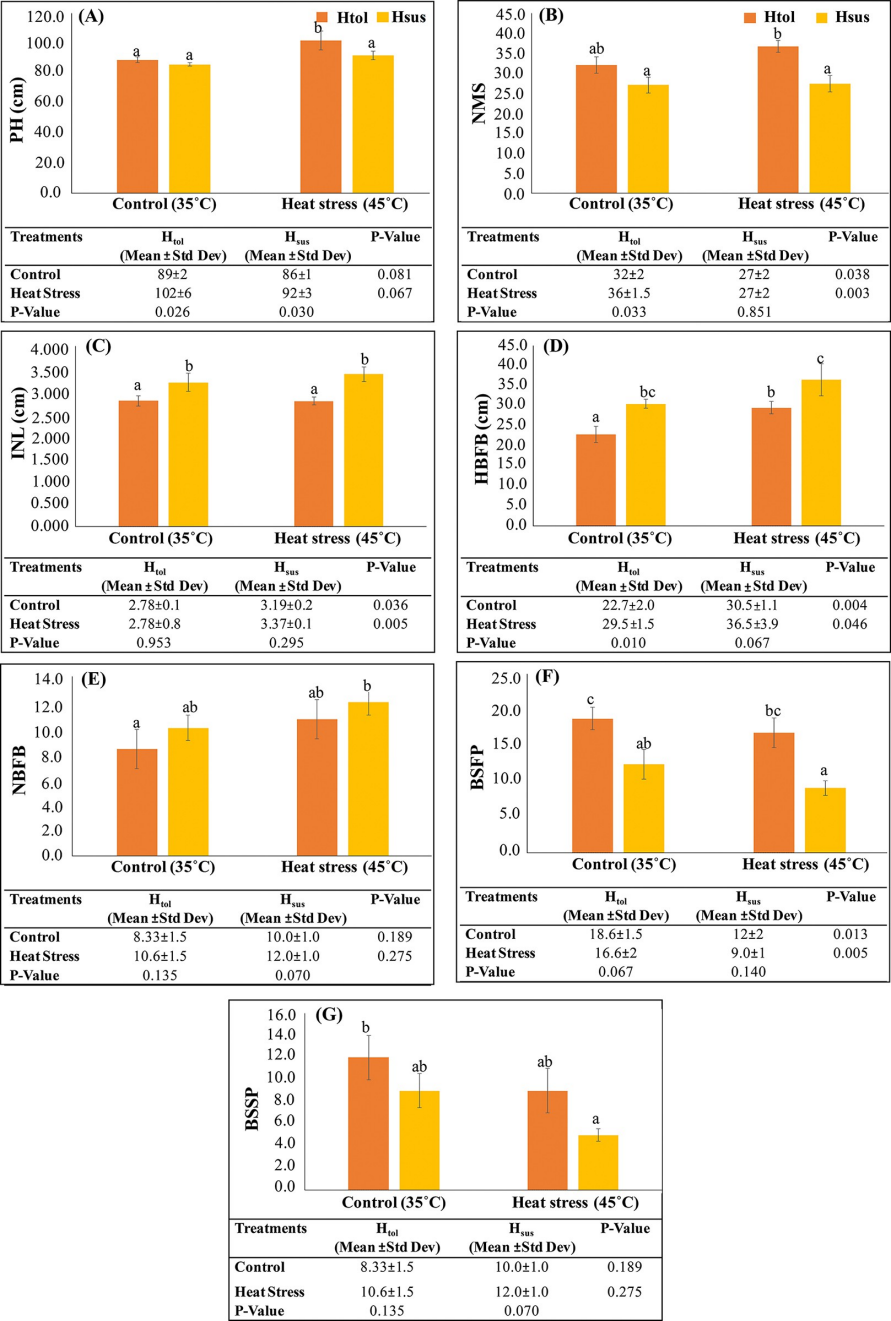
Effect of heat stress on morphological parameters of H_tol_ and H_sus_ genotypes (A) plant height (cm); (B) nodes on the main stem; (C) inter-nodal length (cm); (D) HBFB (cm) (E) NBFB (F) BSFP (G) BSSP. Error bars indicate the mean ± standard deviation of three experimental mean values. P-values of tolerant control and tolerant stress and susceptible control and susceptible stress are given in the table of each section, and *p < 0.05; **p < 0.01; ***p < 0.001 according to ONE-WAY ANOVA. PH = plant height, NMS = nodes on the main stem, INL = inter-nodal length, HBFB = height bearing 1^st^ effective boll, NBFB = node bearing 1^st^ effective boll, BSFP = bolls set in the 1^st^ position; BSSP = bolls set in the 2^nd^ position.

**Fig 3:**
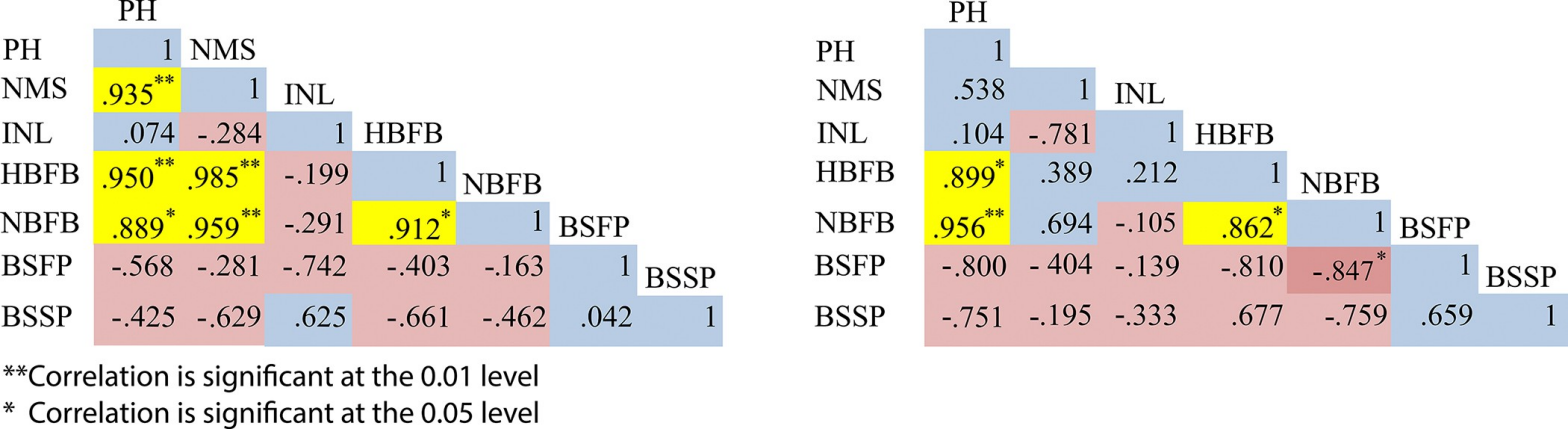
Pearson correlations between different morphological parameters in (A) H_tol_ and (B) H_sus_ genotypes under heat stress. The red squares indicate a negative correlation, the blue squares a positive correlation, and the yellow squares a significant positive correlation.

Different morphological parameters like plant height (PH), nodes on the main stem (NMS), internodal length (INL), height bearing 1st effective boll (HBFB), node bearing 1st effective boll (NBFB), bolls set on 1st position (BSFP), and bolls set on 2nd position (BSSP) in H_tol_ and H_sus_ genotypes were recorded at control and heat stress (Fig 2). H_tol_ and H_sus_ genotypes exhibited a different pattern of morphological changes in plant growth and development under heat stress. The PH increased by 89–102 cm in H_tol_ and 86–92 cm in H_sus_ (Fig 2A), with the related increase in NMS by 32–36 in H_tol_ and 27–27.3 in the H_sus_ genotype at heat stress over control (Fig 2B), and the INL remained the same in H_tol_ and increased by 3.1–3.3 cm in H_sus_ due to the change in PH and NMS (Fig 2C). Compared with control, the HBFB and NBFB increased by 22–29 cm and 8–10 in H_tol_, and 30–36 cm and 10–12 in H_sus_, at heat stress (Fig 2D and 2E). Similarly, the BSFP and BSSP decreased by 18–16, 12–9, and 12–9, 9–5, at stress in the H_tol_ and H_sus_ genotypes, respectively (Fig 2F and 2G). The effect of heat stress was significant at p ≤ 0.05 on all the studied morphological parameters of both genotypes. The PH increased significantly in both Htol and Hsus genotypes with p-values (0.026, 0.030) with the concurrent increase of NMS only in Htol (p = 0.033) under heat stress as shown in Fig 2. Another important parameter, like HBFB, also showed a significant effect under both control and heat stress conditions, with p-values (0.010) showing that the tolerant genotype of cotton retained fruit at lower positions than the susceptible one. Different morphological parameters, like fruit positioning from the lowest node and minimum height and the boll set on the 1^st^ and 2^nd^ position nodes, depicted that the tolerant genotype expressed its resilience to heat stress conditions as an adaptive mechanism as shown in our results (Fig 2F and 2G). These characteristics could be used as a preliminary indicator for the screening of heat tolerant genotypes for breeding programs and molecular studies [36]. The relationship between different morphological parameters of H_tol_ and H_sus_ genotypes in response to heat stress is depicted in Fig 3. Both genotypes exhibited different correlation trends towards morphological traits. In H_tol_, PH was highly significant with NMS and HBFB and significantly correlated with NBFS. NMS is also positively and highly significantly correlated with HBFB and NBFB. HBFB showed significance with NBFB (Fig 3A). In H_sus_, PH is significantly correlated with HBFB and NBFB, while HBFB is significantly correlated with NBFB, and NBFB has a significant negative correlation with BSFP (Fig 3B).

### Physiological traits

**Fig 4:**
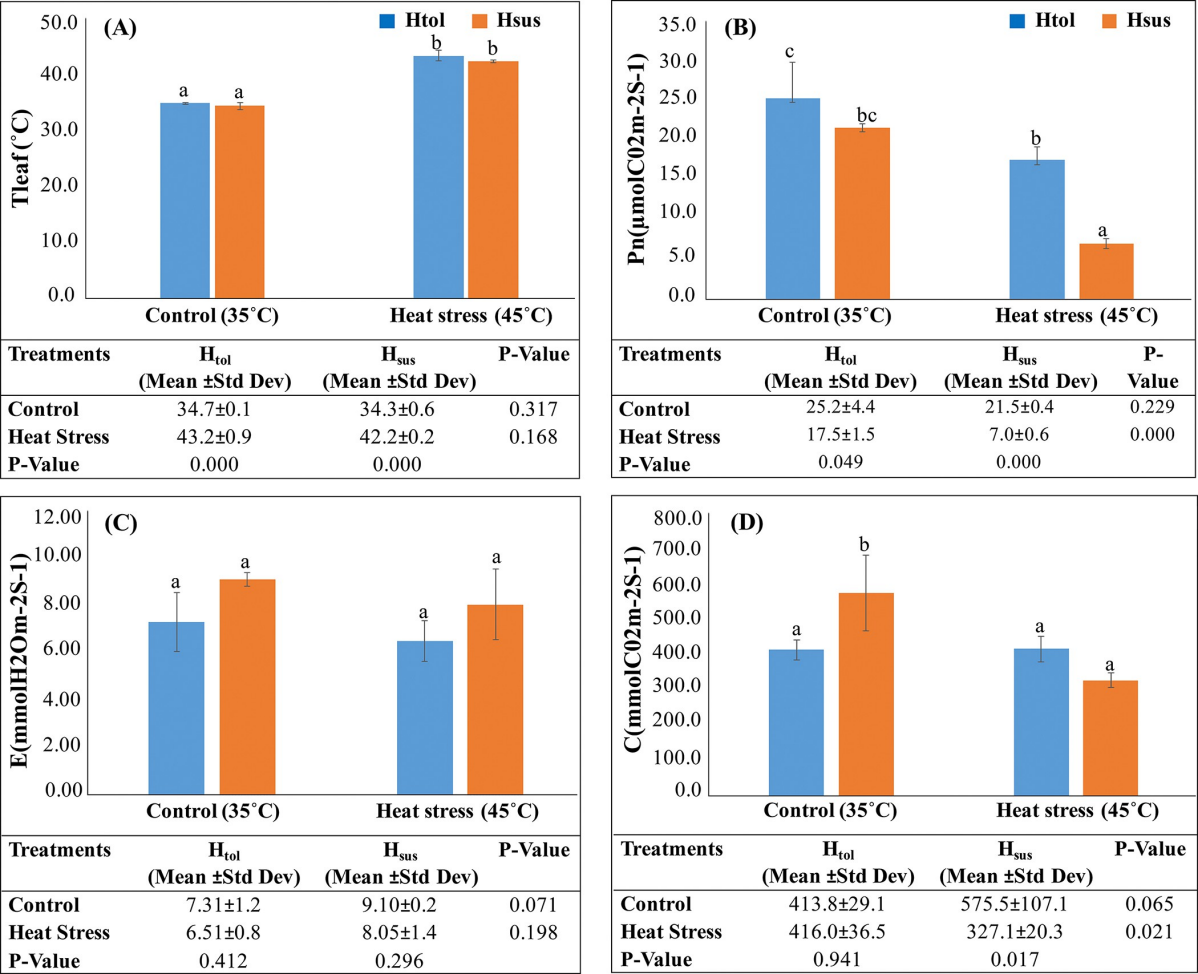
Gas exchange parameters in the leaves of Htol and H_sus_ genotypes in response to heat stress (A) Temperature leaf (°C) (B) Net photosynthetic rate (*Pn*) (C) Transpiration rate (*E*) (D) Stomatal conductance (*C*). Error bars indicate the mean ± standard deviation of three experimental mean values. P-values for tolerant control and tolerant stress and susceptible control and susceptible stress are given in the table of each section and *p < 0.05; **p < 0.01; ***p < 0.001 according to ONE-WAY ANOVA.

Gas exchange characteristics of H_tol_ and H_sus_ genotypes in the control and heat stress were measured from the 3^rd^ to 4^th^ fully expanded leaf with the CI-340 photosynthesis system (Fig 4). Air temperature (T_air_) directly influenced leaf temperature (T_leaf_) (Fig 4A). At heat stress, the H_sus_ genotype exhibited a sharp decline in net photosynthetic rate (*Pn*) (μmolCO_2_m^-2^S^-1^) of 21–7. In comparison to a 25–17 decline in the H_tol_ genotype over control (Fig 4B). Similarly, transpiration rate (*E*) (mmolH_2_Om^-2^S^-1^) was reduced in H_sus_ genotypes by 9.1–8.0 and 7.3–6.5in H_tol_ genotypes over control (Fig 4C). Stomatal conductance (*C*)(mmolCO_2_m^-2^S^-1^) remained high at 575–327 in H_sus_ at control but decreased by 413–416 at stress as compared to H_tol_, where stomatal conductance did not differ significantly (p ≤ 0.05) at heat stress (Fig 4D). Heat stress caused a significant (p ≤ 0.05) impact on all the gas exchange parameters, like *Pn*, with p-values (0.049 and <0.001) of H_tol_ and H_sus_ genotypes, respectively, showing that it was significantly affected by heat stress. Transpiration rate (*E*) increased but did not show significant variations among the genotypes exposed to heat stress, and stomatal conductance was strongly inhibited in H_sus_ (p = 0.017) as compared to H_tol_. Heat tolerance is intimately connected with the capacity of plants to maintain leaf gas exchange and CO_2_ assimilation rates under heat stress and has a significant impact on the intercellular CO_2_ concentration, leaf stomatal conductance (*C*), and leaf water status (*E*), which led to a decline in net photosynthetic rate (Fig 4). Another factor contributing to reduced photosynthesis that impacts intercellular CO_2_ is the closure of stomata. All these parameters collectively contributed to the evaluation of plant thermotolerance under heat stress [37].

### Biochemical responses

**Fig 5:**
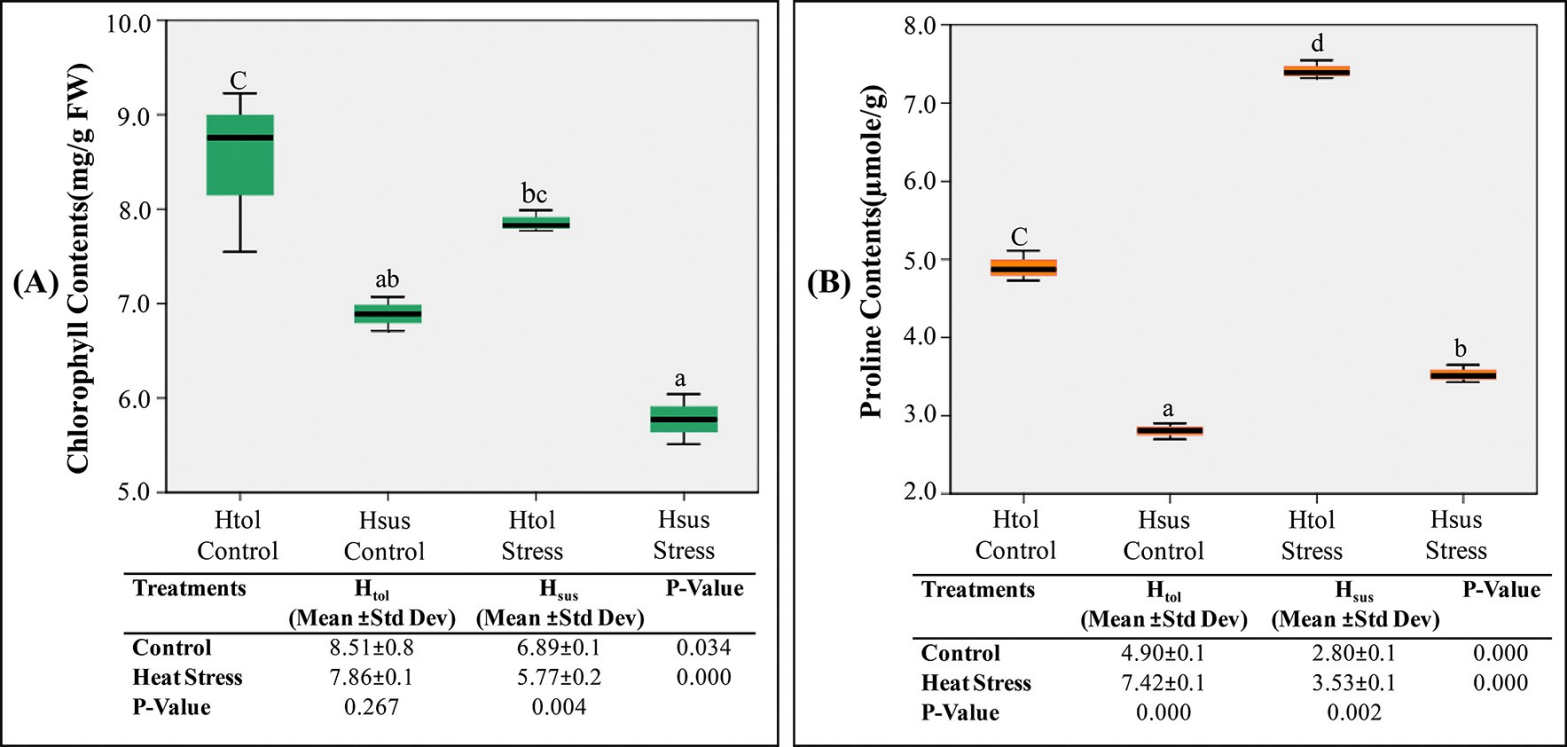
Effect of heat stress on biochemical parameters of H_tol_ and H_sus_ genotypes: (A) chlorophyll concentration (mg/gFW); (B) proline contents (μmoles/g). The box plot showed the upper and lower quartiles with median and whisker lines that extended from the box, showing variability out of the upper and lower quartiles. *p < 0.05; **p < 0.01; ***p < 0.001 according to ONE-WAY ANOVA.

Total chlorophyll_a+b_ was higher in H_tol_ than in the H_sus_ genotype, irrespective of temperature levels (Fig 5A). The elevated temperature lowered chlorophyll_a+b_ contents in both genotypes; however, the reduction in Chl_a+b_ (mg/g FW) was 8.5–7.8 in H_tol_ and 6.8–5.7 in H_sus_ genotype, showing a significant (p<0.05) decline in chlorophyll contents in H_sus_ genotype (p = 0.004) (Fig 5A). At control temperature, the leaf proline content (μmole/g) was 4.9–7.4 higher in H_tol_ as compared to the 2.8–3.5in H_sus_ genotype (Fig 5B). The leaf proline content showed an increasing trend under heat stress; however, the proportionate increase in leaf proline varied greatly in both genotypes. At heat stress, the H_tol_ genotype exhibited the highest proline level of 7.42 *μ*mol g^-1^ FW, while the proline level remained at 3.53 μmol g^-1^ FW in the H_sus_ genotype (Fig 5B). The mean increase in proline accumulation at heat stress over control was higher in the H_tol_ genotype (p<0.001), showing an increase of 51.3%, while the H_sus_ genotype exhibited an increase of 26.0% in leaf proline contents. The chlorophyll content is one of the important indicators used to determine the photosynthetic capacity of plants. Similarly, lipid peroxidation of thylakoid and chloroplast membranes is the factor contributing to the decrease in chlorophyll pigment under heat stress as observed in this study (Fig 5A). The proline contents are intriguing signaling molecules that can help plants to cope with heat stress. The increase in proline contents in the tolerant genotype under stress conditions could be one of the possible adaptation mechanisms for the thermotolerance of plants [38].

### Proteomic analysis of the cotton genotypes at different temperatures

**Fig 6:**
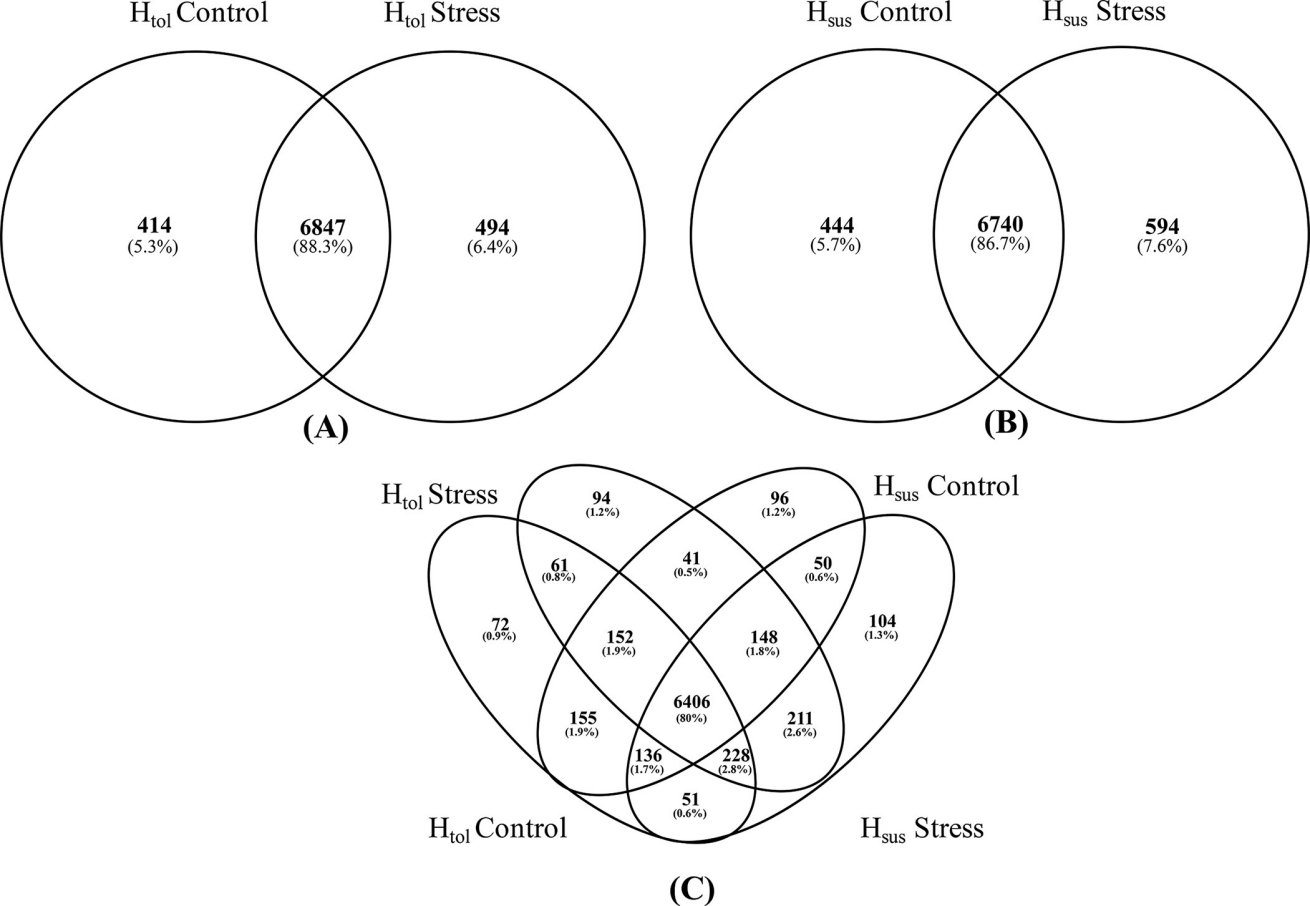
Venn diagram showing the unique and shared proteins of H_tol_ and H_sus_ genotypes in response to heat stress (A) unique and shared proteins of H_tol_ genotypes (B) unique and shared proteins of H_sus_ genotypes (C) comparison of proteins in both genotypes under heat stress.

The identification of proteins was carried out using the proteome discoverer 2.5 [39]. The raw data from nLC-MS/MS at control and heat stress were separately processed. Applying an FDR ≤ 1%, in this analysis, a total of 8005 proteins were identified in leaf tissues, of which 7261 proteins were identified in the H_tol_ genotype at the control and 7341 proteins were present at the heat stress condition (S1 Table and Fig 6). Similarly, in the H_sus_genotype, a total of 7184 proteins were found in the control, and 7334 were present in heat stress (S1 Table and Fig 6). Based on these results, each genotype at the control and heat stress was characterized by its own distinct set of proteins (Fig 6A and 6B), alongside those common between each group at control and heat stress (Fig 6C).

**Fig 7:**
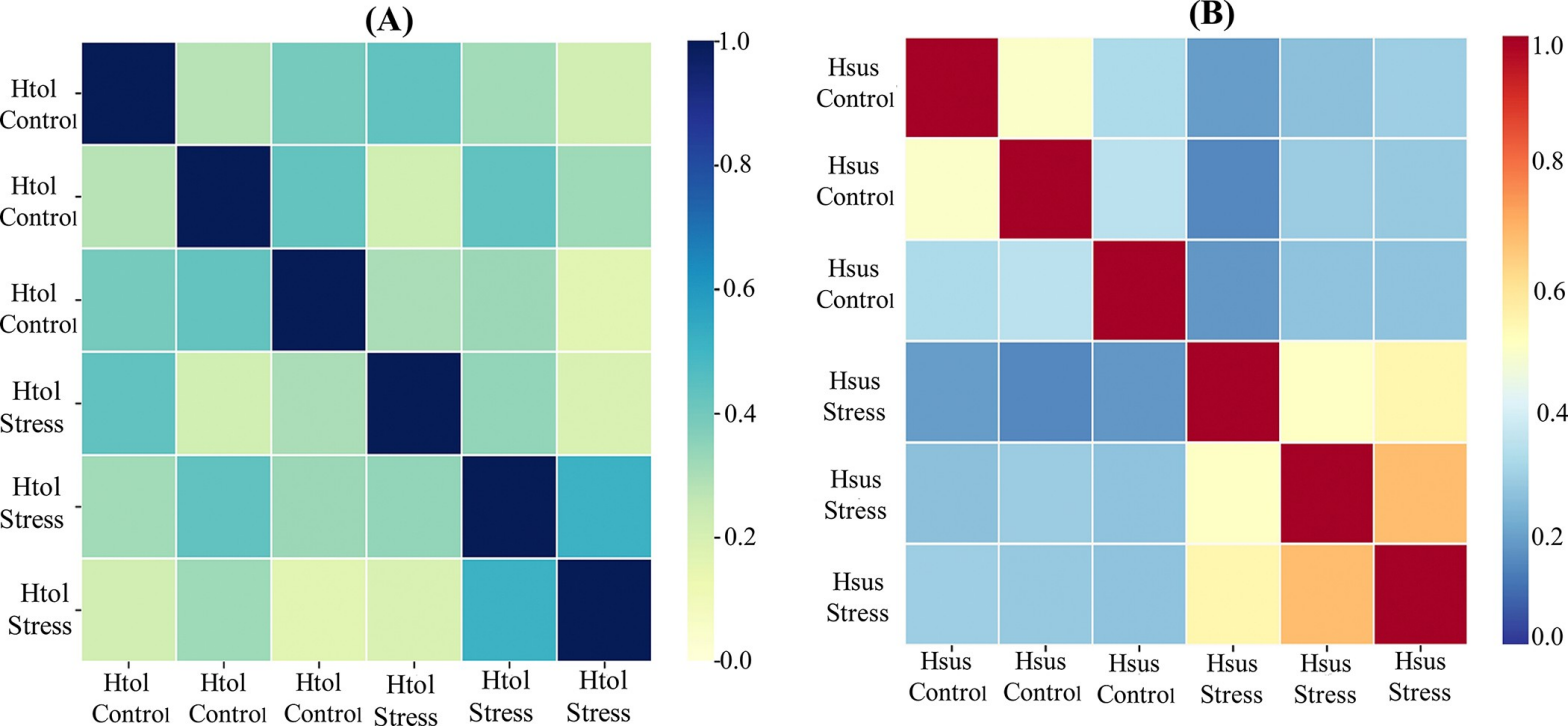
(A) Pearson correlation heatmap of three biological replicates of the H_tol_ genotype (B) Pearson correlation heatmap of three biological replicates of H_sus_ genotypes. The correlation values between ±1 is considered to be a strong correlation.

From Pearson correlation values, the repeatability of the results was evaluated. The results could be reproduced because all of the values were near 1 (Fig 7). Additionally, a histogram was generated to evaluate the distribution of normalized abundance values among the biological replicates of each genotype under control and heat stress. These results demonstrate that the analysis proceeded with good reproducibility. The Pearson correlation heat map for three biological replicates of both genotypes showed significant correlation values near one, which indicates reproducibility, experimental precision, limited biological or technical variability, and carefully controlled experimental conditions (Fig 7A and 7B). Additionally, it ensures that the sample handling procedures, data analyses, and experiment design are well-considered to minimize any potential sources of bias that could accidentally change the correlation results.

**Fig 8:**
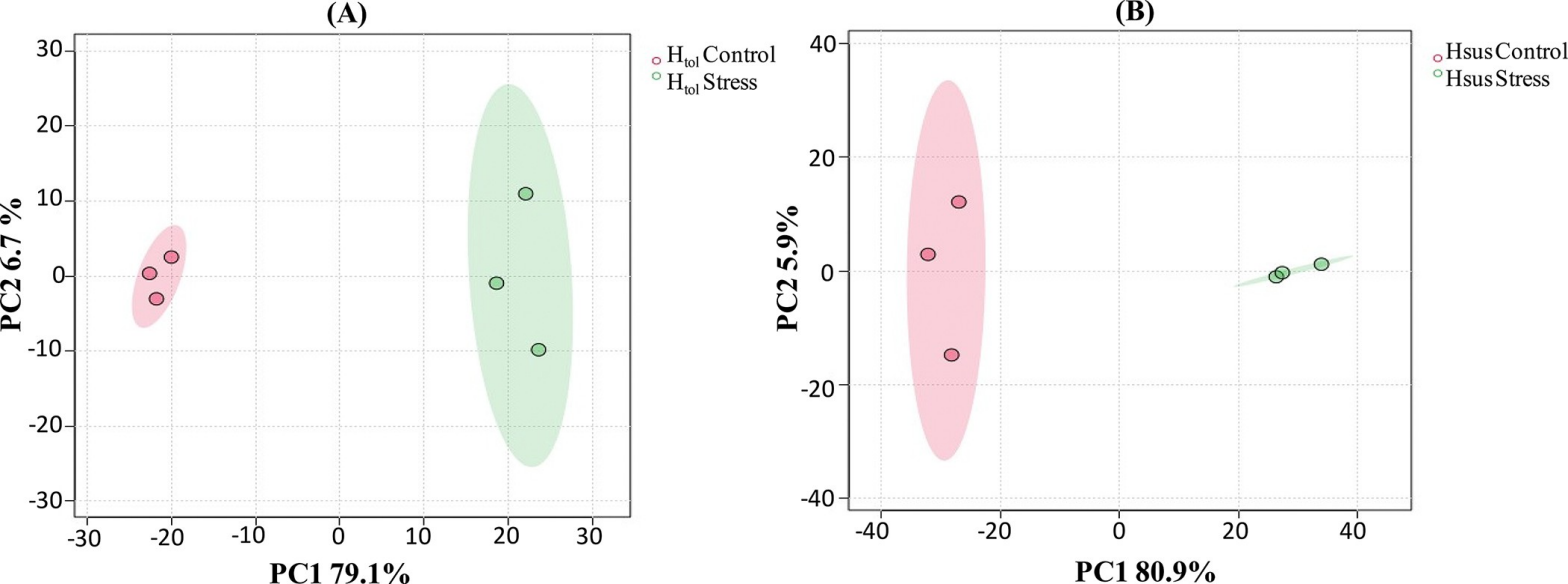
(A) PCA of 3 biological replicates of H_tol_ genotypes at the control and heat stress (B) PCA of 3 biological replicates of H_sus_ genotypes at the control and heat stress.

The principal component analysis (PCA) was used for the differentially expressed proteins identified from the leaves of H_tol_ and H_sus_ genotypes at the control and heat stress conditions. PCA effectively indicated the distinct accumulation pattern of these proteins on a 2-dimensional graph based on their abundance similarities (Fig 8A and 8B). PC1 represented more variation in the data as compared to PC2, which indicates that the impact of heat stress on the two genotypes is higher compared to the variation between the genotypes themselves. Similarly, the variation between PC1 values of the H_tol_ (79.1%) and H_sus_ (80.9%) genotypes revealed that the influence of heat stress was higher on both the tolerant and susceptible plants, while the PC2 values of 6.7% and 5.9% reflected a low genetic variation among the genotypes.

### Significantly differentially abundant proteins

**Fig 9:**
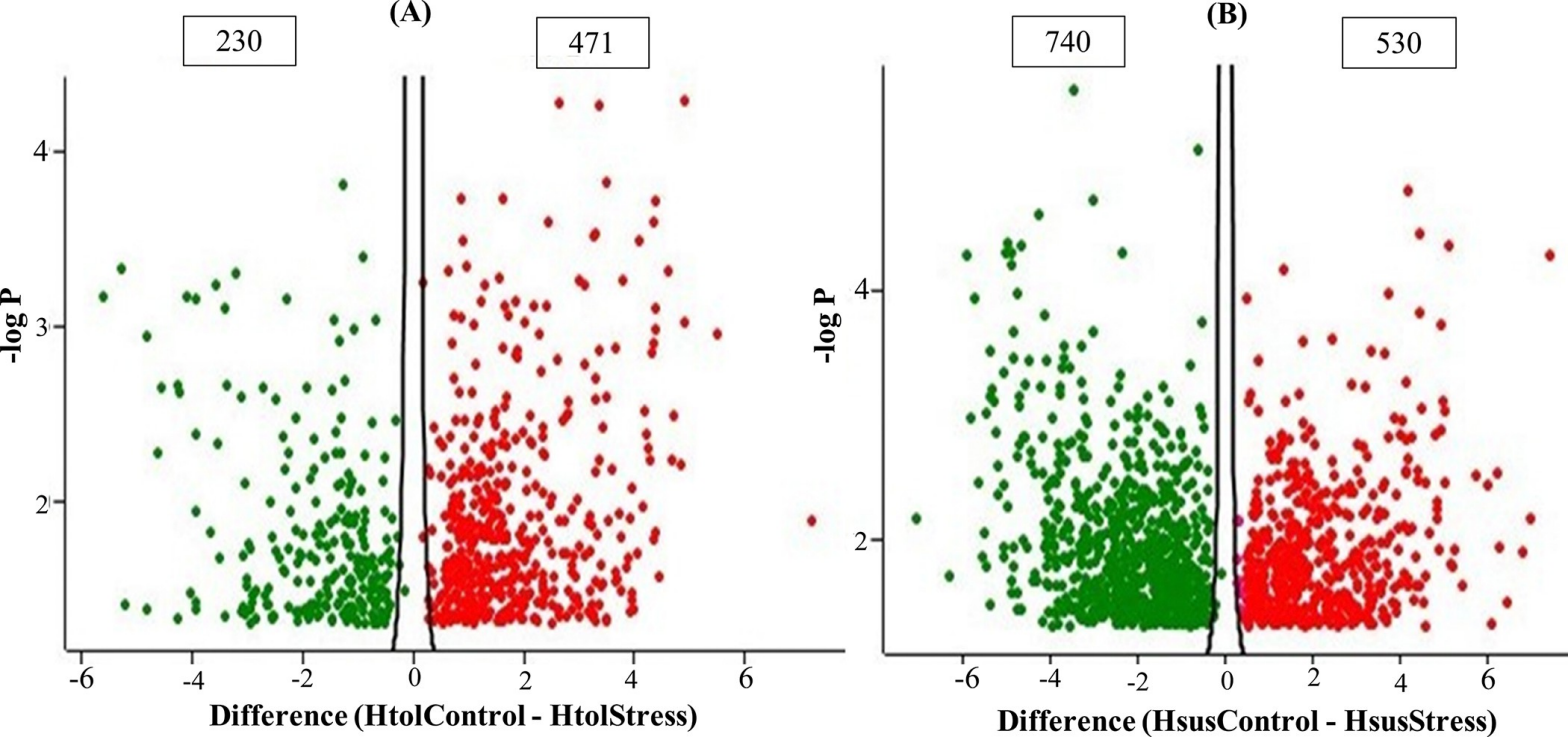
(A) Volcano plot of 3 biological replicates of H_tol_ genotypes at control and heat stress (B) A volcano plot of three biological replicates of H_sus_ genotypes at the control and heat stress. Each point represents a protein with a difference between the abundance of each genotype at control and stress along the x-axis and the log10 p-value along the y-axis. The red and green points represent the upregulation and downregulation of proteins, respectively.

The T-test was applied to identify proteins with significant differences between each genotype under control and heat stress. The 701 proteins were differentially expressed (DEP) at p ≤ 0.05 in the H_tol_ genotype (S2 Table and Fig 9A). Of these, a total of 230 proteins (32.81%) were down-regulated and 471 (67.1%) were up-regulated at control and stress, respectively. In the H_sus_ genotype, a total of 1270 proteins were differentially expressed (p ≤ 0.05) at control and heat stress (S3 Table and Fig 9B). Among 1270 DEPs, 740 proteins (58.2%) were downregulated and 530 (41.7%) were upregulated at control and stress, respectively. The impact of high temperatures was diverse across both. The significant change in proteins was higher in the H_sus_ genotype at stress compared to the H_tol_ genotype.

### Functional analysis of differentially expressed proteins

**Fig 10:**
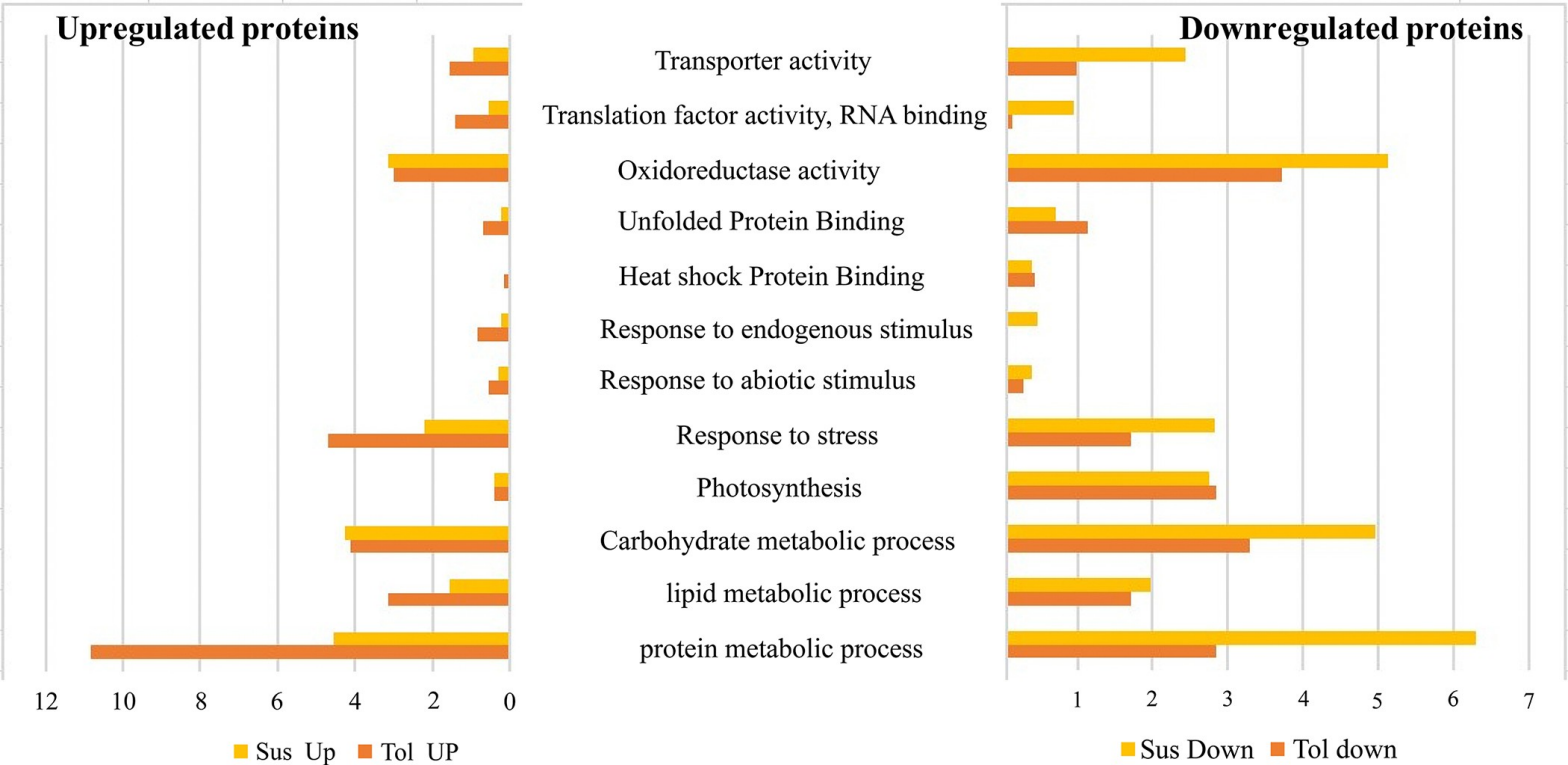
Selected functional classes of the significantly differentially expressed proteins under heat stress. The bars illustrate the percentage of each class concerning total differentially expressed proteins. The orange color showed the tolerant plant and the yellow susceptible genotypes, as indicated.

To gather information regarding the functions, the DEPs proteins of both genotypes were subjected to functional classification through gene ontology (GO) using the AgBase database and tool (https://agbase.arizona.edu/index.html). In the H_tol_ genotype, the down-regulated proteins were classified into 35 categories of GO biological process (GOBP), 19 categories of GO molecular function (GOMF) annotation, and 20 categories of GO cellular component (GOCC) (S4 Table), while the up-regulated proteins were classified into 37 categories of GOBP, 21 categories of GOMF annotation, and 22 categories of GOCC (S5 Table). Similarly, in the H_sus_ genotype, the down-regulated proteins were classified into 36 categories of GOBP, 23 categories of GOMF annotation, and 24 categories of GOCC (S6 Table), while the up-regulated proteins were classified into 38 categories of GOBP, 21 categories of GOMF annotation, and 23 categories of GOCC (S7 Table). The major biological and molecular processes that respond contrarily to heat stress in both genotypes include protein metabolic processes, oxidoreductase activity, photosynthesis, response to stresses, abiotic stresses, endogenous stimulus, HSPs, unfolded protein binding, translation, and transporter activities (Fig 10). The majority of proteins were diversely regulated upon heat stress related to the protein metabolic process, and a low number of proteins were downregulated (2.8%), and more were upregulated (10.8%) in the H_tol_ genotype as compared to the H_sus_ genotype (Fig 10). The ROS-scavenging proteins associated with cellular response to oxidative stress, like glutathione peroxidase, catalase, and superoxide dismutase, exhibited downregulation in both genotypes, while BRISC and BRCA1-A complex proteins were upregulated in the H_tol_ genotype, which showed downregulation in the H_sus_ genotype (Table 1 and Fig 10). Many proteins associated with responses to stresses like abiotic stresses and endogenous stimuli undergo upregulation under heat stress. The most intriguing finding regarding the response to the endogenous stimulus was the presence of MLP-like protein 423 that upregulated in the H_tol_ genotype while, in the H_sus_ genotype, many proteins were downregulated, contrary to the H_tol_ genotype (S6 Table). Another apparent difference is the lesser number of stress-responsive proteins that endure the downregulation in the H_tol_ genotype. Only one protein, namely luminal-binding protein 5, was upregulated in the tolerant genotype as compared to the susceptible genotype, where none of the proteins was upregulated under heat stress (S5 and S6 Tables). It is important to mention that in both genotypes, the photosynthesis-associated proteins were downregulated under heat stress, including chlorophyll-binding proteins, photosystem I and II reaction systems, and oxygen-evolving enhancer proteins. While a few proteins were upregulated in response to heat stress, they were involved in the oxidative photosynthetic carbon pathway and electron transport chain. The important protein phosphoribokinase associated with the Calvin cycle was downregulated in the H_sus_ genotype, while the upregulation of (S)-2-hydroxy-acid oxidase was shown in the H_tol_ genotype under heat stress (S5 and S6 Tables). Eleven proteins were involved in transmembrane transporter activity, like ABC type, voltage-gated anion, P-type calcium transporter, and proton transporter. ATP synthesis was found to be upregulated in the H_tol_ genotype, while a significant decrease in protein regulation was observed in the H_sus_ genotype in the proteins related to transport function. Specifically, in the H_tol_ genotype, our results also indicated the presence of a significant primary active transporter, namely NADH dehydrogenase subunit 9 (NADH ubiquinone oxidoreductase or complex I) (S5 Table). Beta-glucosidase BoGH3B-like was among the proteins in the functional category of carbohydrate metabolism that were solely impacted by heat stress in the H_sus_ genotype while upregulated in the H_tol_ genotype (S5 and S6 Tables).

**Table 1:** Selected significantly differentially expressed proteins between the tolerant and susceptible cotton genotypes.

Sr. No.	Uniprot IDs	Protein name	H_tol_Up/Down	H_sus_Up/Down	H_tol_ P-values	H_sus_ P-values	Htol Abundance	Hsus Abundance
Protein metabolic process								
1	A0A1U8IPL0	glycine—tRNA ligase	-	-	1.45	2.84	-0.611	-1.026
2	A0A1U8LGL0	SBT2.5	+	+	1.547	1.476	2.188	2.078
3	A0A1U8KQS9	RD21a	+	+	1.452	1.554	2.908	3.383
4	A0A1U8JYE7	mitochondrial-processing peptidase	+	+	1.352	2.648	1.016	1.203
5	A0A1U8IP15	Lysine-tRNA ligase	+	+	1.452	1.724	1.603	2.932
6	A0A1U8NWX7	threonine-tRNA ligase	+	+	3.486	1.931	0.899	1.162
7	A0A1U8KVY4	Ubiquitin receptor RAD23	+	+	1.808	2.323	1.701	1.031
8	A0A1U8MF38	E1 ubiquitin-activating enzyme	+	+	1.314	1.36	0.67	0.851
9	A0A1U8KZF0	eukaryotic translation initiation factor 4B2	+	+	2.593	2.608	1.691	1.615
10	A0A1U8NPN1	protein BOLA2	+	+	1.537	2.168	3.293	4.855
11	A0A1U8I0E3	exosome complex component RRP45B	+	+	1.36	1.745	1.751	1.858
12	A0A1U8K553	Glycine cleavage system H protein	+	+	1.838	2.066	0.865	0.954
Carbohydrates metabolic process								
1	A0A1U8J484	beta-glucosidase BoGH3B	+	-	2.737	1.37	2.313	-0.934
2	A0A1U8M5M9	glycosyltransferase-like KOBITO 1	+	+	1.514	1.357	2.359	2.03
3	A0A1U8PEU7	Inositol-1-monophosphatase	+	+	1.54	2.194	0.761	1.104
5	A0A1U8NSA4	phosphoglycerate mutase	+	+	1.879	2.486	1.005	0.747
Photosynthesis								
1	A0A1U8HQH5	Mg-protoporphyrin IX chelatase	+	NA	2.154	NA	0.31	NA
2	A0A1U8JF27	photosynthetic NDH subunit of subcomplex B 1	+	NA	1.53	NA	1.224	NA
3	A0A1U8M0A9	(S)-2-hydroxy-acid oxidase	+	NA	1.863	NA	1.185	NA
4	A0A1U8MY22	Phosphoribulokinase	NA	+	NA	2.075	NA	-0.386
Stress Responsive proteins								
1	A0A1U8KR45	BRISC and BRCA1-A complex	+	-	1.599	1.636	3.645	-3.876
2	A0A1U8JWS2	luminal-binding protein 5	+	-	2.183	1.346	0.263	-0.873
3	D2D328	MLP-like protein 423	+	NA	1.301	NA	1.101	NA
Oxidoreductase activity								
1	A0A1U8LQ46	secoisolariciresinol dehydrogenase	+	-	2.852	2.275	4.318	-3.268
2	A0A1U8N366	1-aminocyclopropane-1-carboxylate oxidase	+	-	3.116	2.145	2.188	-1.675
Transporter Activity								
1	S4SJL9	NADH dehydrogenase subunit 9	+	NA	2.429	NA	1.484	NA
2	A0A1U8NH34	aquaporin PIP2-2	+	NA	2.535	NA	1.647	NA
3	A0A1U8IL78	aquaporin TIP1-3	+	NA	1.487	NA	3.208	NA
4	A0A1U8L877	ERD6-6	+	NA	1.365	NA	3.908	NA

**Fig 11:**
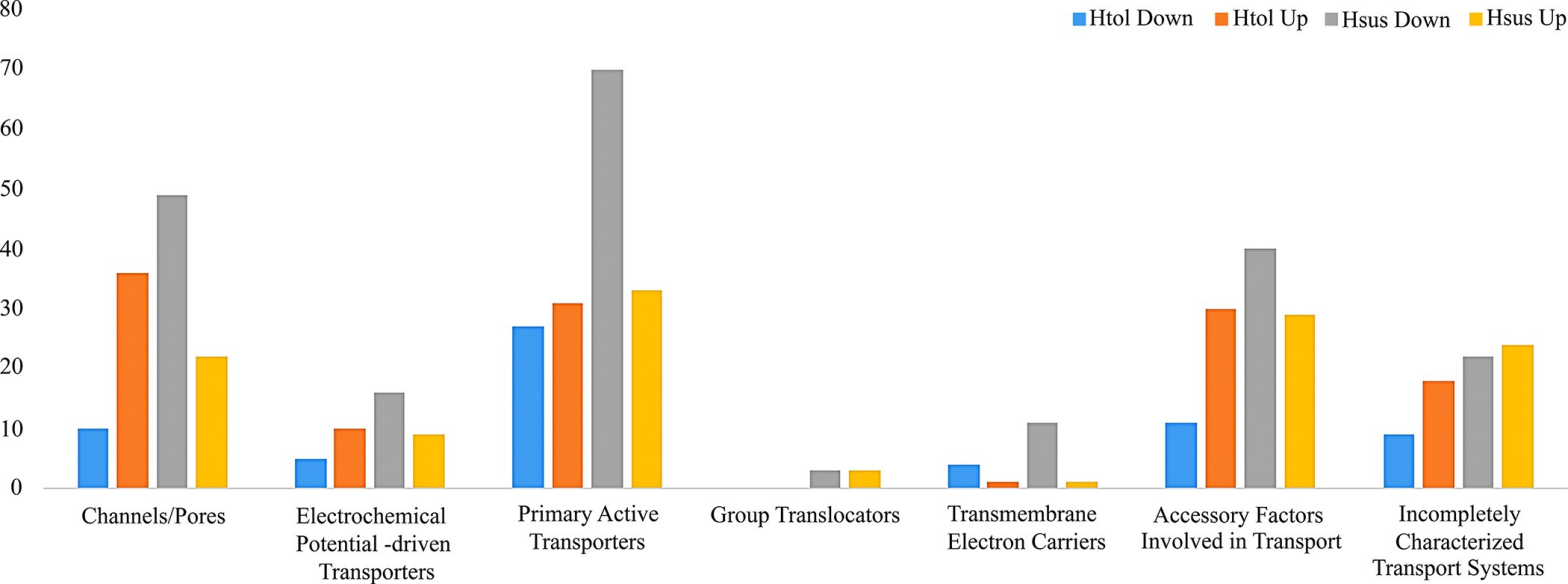
Various classes of transporter proteins were identified from the leaves of H_tol_ and H_sus_ genotypes in response to heat stress following the Transporter Classification Database (TCDB).

In addition to other significant proteins involved in various mechanisms, we identified differentially abundant proteins related to transport. According to the Gene Ontology (GO) classification, most of the DEPs in this class faced downregulation in the H_sus_ genotype under heat stress (S6 Table). These proteins are categorized into seven groups according to the TCDB database (Fig 11).

## Discussion

An integrated morpho-physiological, biochemical, and proteomic strategy was used to analyze the two locally developed genotypes, namely H_tol_ and H_sus_. Morphological analysis and statistical correlation revealed that the H_tol_ genotype exhibited greater resistance to heat stress compared to the H_sus_ genotype, as shown in Figs 1 and 2. It was previously reported that the severity and length of exposure to temperature stress reduced the plant height, sympodial branches, internodes, seeds per boll, boll weight, monopodial branches, and fiber length, which ultimately have an impact on the reproductive and vegetative phase of the plant [40]. The net photosynthetic rate is an important factor for normal cotton crop output and boll retention, but high temperatures limit photosynthesis by enhancing the CO_2_ supply between intercellular spaces and sites of carboxylation [10]. The morphological and physiological findings in this study demonstrated that H_tol_ genotypes exhibit higher control over H_sus_ for managing stomatal conduction, transpiration rate, and net photosynthetic rate as shown in Fig 3. Stomatal conductance is essential for managing water loss and photosynthesis efficiency during heat stress, and high-temperature tolerance depends on maintaining photosynthetic activity and regulating stomatal behavior [41]. Our results align with previous findings that high temperatures inhibit the stomatal conductance (*C*) and net photosynthetic rate (*Pn*) [10]. This ability to maintain photosynthetic activity under heat stress contributes to improved boll retention and overall crop yield, making the Htol genotype more advantageous for cultivation under high-temperature conditions.

The genetic variations in the H_tol_ genotype preserve the assimilation of photosynthetic pigments (chlorophyll contents) per leaf as seen in Fig 4A, as brought out by the thickness of the leaves. High temperatures have been associated with damage to photochemical processes in thylakoid lamellae and carbon metabolism in the stroma of the chloroplast [42]. Proline accumulation under heat stress is known to stabilize cellular osmotic potential and scavenge reactive oxygen species, which offers critical biochemical support to plants in abiotic stress. In cotton, elevated proline levels are closely associated with stress-tolerant genotypes that maintain both physiological and antioxidant functions [43]. Under heat stress, less accumulation of proline in H_sus_ genotype makes it more susceptible to changing environments and is endorsed by the previous findings that proline contents and heat tolerance were strongly correlated with genotypes’ genetic potential with higher enzymatic activity, water use efficiency, and physio-biochemical characteristics [44].

Accumulation of proline regulates the overall changes in the stomatal closure, transpiration rate, and net photosynthetic rate and causes the proteolytic degradation of many proteins that are misfolded and denatured in stressed cells [5]. Significant metabolic alterations are required to preserve cell homeostasis during stress, and these alterations may be partially achieved by altering the expression of essential proteins [4].

Proteomic analysis identified differentially expressed proteins linked to stress response, as well as those involved in carbohydrate, protein, and lipid metabolism; oxidoreductase activity; transporter function; and translation. The H_tol_genotype showed a significant variation in the abundance of the proteins related to these mentioned pathways. The upregulation of stress-responsive proteins such as heat shock proteins and ubiquitin enzymes in the Htol genotype indicates a robust proteostasis system that preserves protein quality and prevents leaf senescence, which was not observed in the Hsus genotype.

### Effect of heat stress on protein metabolism

In response to heat stress, out of 701 DEP, 74 were upregulated in the H_tol_ genotype (S2 Table) and 57 in the H_sus_ genotype (S3 Table); uniquely, only one protein was downregulated, and eleven proteins were upregulated, which are present commonly in both genotypes. These upregulated proteins were mostly found to have functions in translation, glycosylation, lipoylation, and proteolysis.

Heat stress in cotton plants enhances the accumulation of misfolded and denatured proteins and decreases the stability of mRNA and ribosomal RNA to synthesize the important proteins [45]. Glycine-tRNA ligase was used for stabilizing the protein synthesis by charging the tRNA with glycine during translation and was the only specific protein that was shown to be down regulated in our study in both genotypes (Table 1). Our research findings are supported by the previous study on tobacco plants where glycine-tRNA ligase becomes upregulated in tolerant genotypes under heat stress conditions [46] Similarly, glycine-tRNA ligase plays a role in sorghum seedlings by supporting tolerance mechanisms under heat stress [47].

Out of 11 proteins that were upregulated in both genotypes under heat stress, three (RD21a, SBT 2.5, and mitochondrial-processing peptidase) belong to serine, cysteine, and metallo peptidases (Table 1). They play a critical role in protein proteolysis in the chloroplast, mitochondria, and nuclei of the leaves and thereby prevent the accumulation of protein aggregates [48–50]. These peptidases assist in nutrient recycling because they regulate protein levels and support crop yield. Ubiquitin-mediated proteasome degradation is essential for eliminating misfolded proteins and supporting plant tolerance under heat stress. The selective removal of these proteins through the ubiquitin-proteasome pathway maintains cellular homeostasis and prevents protein aggregation, which enhances stress tolerance [51]. The upregulation of these three proteases in our study indicated these proteins play an essential role in protein regulation in the presence of heat stress, which ultimately protects from leaf senescence and may lead to other tolerance mechanisms [52].

Two proteins, namely lysine-tRNA ligase and threonine-tRNA ligase, are associated with the tRNA aminoacylation for protein translation and were found upregulated under stress in our study, and previous research showed that these play a vital role in the proper folding of the heat shock proteins during stressful conditions [53,54] (Table 1). Under heat stress, the synthesis of heat shock proteins is vital for the survival of plants [55]. In our findings, to increase the tolerance in plants under heat stress, these two proteins become upregulated, which assists in the synthesis and appropriate folding of heat shock proteins. Heat stress activates the protein degradation pathways like the ubiquitin-proteasome system (UPS) and autophagy to remove damaged and unfolded proteins [56]. Two other proteins that were upregulated in H_tol_genotypes included the ubiquitin receptor radiation-sensitive protein (RAD23) and the E1 ubiquitin-activating enzyme (Table 1). These two proteins are related through the ubiquitination process and play a crucial role in nucleotide damage repair. The ubiquitination process targets the misfolded proteins for degradation, ensuring cell survival and function during stressful conditions. Ubiquitin ligases, like AtPPRT1 in Arabidopsis, enhance thermotolerance by mediating the degradation of specific stress-related proteins, which highlights the importance of the UPS in plant stress responses [57]. Moreover, in response to heat stress, the mechanisms of nucleotide damage repair are inhibited. Recent studies have revealed that E3 ubiquitin ligases play a role not only in proteostasis but also in DNA damage repair during stress. These mechanisms ensure the degradation of malfunctioning proteins and facilitate nucleotide repair, critical for maintaining cellular functions under stress [58]. The upregulation indicates that they might be overcoming this inhibition, as reported previously [59,60]. Whereas, RAD23 was also previously found to be upregulated in biotic or abiotic stressors (drought) in cotton leaves [61]. All these previous findings endorsed our results that the upregulation of these proteins might participate in the tolerant genotype to stabilize the cellular homeostasis under stress conditions.

Another upregulated protein was eukaryotic translation initiation factor 4B2/eIF4B2 (Table 1). This suggests a potential involvement of eIF4B2 in the translation of HSPs. They are crucial for mitigating the detrimental effects of heat-induced damage in cells [55]. Similar effects might have been caused by the other upregulated protein, namely protein BOLA2, which participates in protein maturation (Table 1). These proteins have possibly played a role in oxidative stress conditions through the activation of antioxidant enzymes [62]. These findings highlight that breeding programs can improve cotton heat tolerance by selecting genotypes with enhanced antioxidant enzyme activity, efficient proteostasis, and sustained photosynthetic capacity. Another important role of protein BOLA2 is associated with molecular chaperone activity, and it is noteworthy that most members of HSPs also perform significant chaperone functions.

Exosome complex component RRP45B was also found to be upregulated under heat stress (Table 1). Although the direct role of this exosome complex component has not been associated with heat stress, the expression of exosomes is altered in response to heat stress and other environmental stressors [63]. The upregulation of this exosome complex component in the H_tol_ genotype in our study indicates its importance in heat stress responses.

Another important protein that increased in abundance was the glycine cleavage system H protein (GCS) (Table 1). Under high-temperature stress and drought conditions, C3 plants simultaneously regulate photorespiration and photosynthesis via feedback inhibition of the glycine to serine conversion phase, and the glycine cleavage system plays a significant role in this network [64]. The upregulation of this protein in the H_tol_ genotype as compared to H_sus_ strongly emphasized the role of this protein to maintain the photosynthetic rate in the former. This further supports our physiological results to maintain the net photosynthetic rate (*Pn*) in H_tol_ genotype under heat stress regarding previous studies.

The upregulation of various proteins in protein metabolism in response to heat stress in the H_tol_ plant indicates that the plant copes with elevated temperatures through different mechanisms at the same time. It can increase the synthesis of HSPs at the translation phase or the maturation phase. These processes might be supported by the inhibition of the aggregation of certain proteins that might have caused leaf senescence. Moreover, ubiquitination also has a significant role in cell survival under stress conditions, as indicated by the upregulation of two proteins that primarily perform ubiquitination. Conversely, some upregulated proteins, such as RAD23, are directly involved in the response to heat stress.

### Proteins related to carbohydrate metabolic processes

Like the protein metabolic process, high temperature also affects the carbohydrate metabolic pathways, mainly the proteins participating in glycolysis, gluconeogenesis, sucrose, starch, pectin, cellulose biosynthesis, and other carbohydrates anabolic and catabolic processes. A total of 63 DEPs were downregulated in the H_sus_ genotype (S3 Table) as compared to 23 DEPs in the H_tol_ genotypes (S2 Table) under heat stress conditions. The downregulation of DEP was significantly greater in the H_sus_ genotype as compared to the H_tol_ genotype, revealing the sensitivity of the genotype to high-temperature stress. Among both genotypes, beta-glucosidase (A0A1U8J484), i.e., BoGH3B-like, is the prominent enzyme that was found to be upregulated only in the H_tol_genotype and downregulated in the H_sus_ genotype (Table 1). Beta-glucosidases are important enzymes that participate in many functions, including lignification, defense, secondary metabolism, symbiosis, cell wall catabolism, signaling, phytohormone conjugate activation, and scent release in plants [65]. According to previous studies, beta-glucosidases play a critical role in plant growth and development under biotic and abiotic stress conditions [66]. They are not only vital in carbohydrate metabolism but also play key roles in stress responses by regulating sugar signaling pathways and releasing active hormones such as abscisic acid to enhance tolerance [67]. Under heat stress, plants shift carbohydrates allocation from growth towards survival processes. For instance, sugars may be directed towards the synthesis of compatible solutes (proline and trehalose) that help in osmo-protection. BoGH3B- like enzymes can hydrolyze inactive ABA-glucose esters (ABA-GE) into free ABA to allow the plants a better adaptation to different stress conditions, including temperature, salinity, and drought [68] and this can be verified by an increase in proline contents of H_tol_ in our biochemical studies as shown in Fig 4B. Our biochemical and proteomic findings revealed that both proline contents and increased abundance of beta-glucosidase in the H_tol_ genotype are in line with the increase in plant growth and development in comparison with the H_sus_ genotype.

A total of four common proteins (glycosyltransferase-like KOBITO-1, inositol-1-monophosphatase, malate dehydrogenase (MDH), and phosphoglycerate mutase (2,3-diphosphoglycerate) were identified as upregulated in both genotypes under heat stress (Table 1). Malate dehydrogenase (MDH) and phosphoglycerate mutase (2,3-diphosphoglycerate) are involved in the TCA cycle and glycolysis pathway. Both enzymes play a vital role in cellular redox homeostasis, the production of energy, and plant growth and development under abiotic stresses. Different research studies revealed that cotton plants maintained their development under nutrient deficiency and aluminum stress by changing MDH expression [69] and changes in this protein expression in our study exhibited that both genotypes tend to maintain their cellular homeostasis. Similarly, in wheat-tolerant genotypes, the increased concentration of phosphoglycerate mutase contributed to the regulation of cell death and oxidative stress and improved resistance to drought stress [70].

Glycosyltransferase-like KOBITO and inositol-1-monophosphatase both were found to contribute to the cell wall and plasma membrane functions. Both regulate the biosynthesis of cell wall components such as cellulose, hemicellulose, and pectin while maintaining cell wall integrity and functionality under salt stress [71]. KOBITO1 may indirectly participate in the glycosylation of ABA signaling components like ABA receptors, which affect the stability, activity, and subcellular localization of proteins. ABA signaling is interconnected with the osmotic regulation and antioxidant defense of tolerant plants to cope with changing environmental conditions. So, the increased abundance of both enzymes in our findings, glycosyltransferase and Beta-glucosidase participate in cell wall lignification and stabilize the cell membrane integrity for the transport of osmolytes to maintain the cellular redox balance. Additionally, in response to salt stress, glycosyltransferase-like KOBITO activity was particularly downregulated in the root epidermis and cortex with the reduced activity of ABA [72]. The inositol-1 monophosphatase (IMPase) was another enzyme that was upregulated in H_tol_ leaves and according to previous studies was involved in signal transduction, membrane integrity, osmoregulation, modulation of ion channels, and other stress responses [73]. In the current study, the increase in abundance of inositol phosphatases in tolerant leaves regulates the said functions by maintaining the inositol metabolism and assisting in the adaptation of plants to stress conditions. The stress-induced increased accumulation of the IMPase enzyme via the ABA signaling pathway has also been noticed in chickpeas, where it regulates the inositol metabolism under drought stress and plays a significant role in the tolerance mechanism [74].

Most of the proteins involved in leaf carbohydrate metabolism are associated with energy production, maintaining cellular redox homeostasis through ABA signaling to regulate the osmotic potential of tolerant plants to withstand stress conditions. Beta-glucosidases and glycosyltransferase-like KOBITO were both upregulated, showing their significant participation in cell wall and cell membrane integrity and thereby supporting the overall growth and development of the H_tol_ genotype under heat stress.

### Response of photosynthesis-related proteins to heat stress

Photosynthesis is the main function, which is distressed by the high temperature, and a significant percentage of DEPs are linked to it. About 2.8% and 2.7% of proteins confronted downregulation, while only 0.42% and 0.3% have shown upregulation upon heat stress in H_tol_ and H_sus_ genotypes, respectively (S5 and S6 Tables). Gene ontology functional analysis exhibited that three proteins, Mg-protoporphyrin IX chelatase, photosynthetic NDH subunit of subcomplex B1, and (S)-2-hydroxy-acid oxidase, were upregulated in H_tol_ genotypes in response to heat stress and mainly contributed to the photosynthetic pathway (Table 1). One of them is Mg-protoporphyrin IX chelatase, which is a potential signaling molecule involved in photosynthesis that inserts Mg^2+^ into protoporphyrin that accumulates in chloroplasts during stressful situations, regulates the antioxidant defense system, and helps to scavenge ROS and thereby maintain chlorophyll integrity [75]. It has been reported [76] that the Mg-protoporphyrin IX chelatase significantly controls plant tolerance to environmental stressors by enhancing stress-responsive proteins’ production under increased light and herbicide stresses. Related to previous studies, the biochemical and proteomic investigation of H_tol_ leaves showed the increased abundance of Mg-protoporphyrin IX chelatase that assists in the maintenance of chlorophyll contents and ultimately increases the *Pn* in the H_tol_ genotype, which decreases in the H_sus_ genotype under heat stress as shown in Figs 3B and 4A.

According to the proteomic analysis, the upregulation of photosynthetic proteins is greater in the H_tol_ genotype as compared to the H_sus_ genotype under heat stress. Another important protein was the photosynthetic NDH subunit of subcomplex B1, which plays a critical role in facilitating the cyclic electron flow (CEF) around PSI, which generates ATP without the production of NADPH under stress conditions when Calvin cycle demand for NADPH decreases due to limited CO_2_ availability due to stomatal closure [77]. These proteomic outcomes are also in line with the preserving of chlorophyll contents and *Pn* values by H_tol_ in the physiological and biochemical investigation of our study. It indicates that the increased abundance of NADH complex protein in H_tol_ genotype reduces the risk of excess electron accumulation at PSI, preventing ROS production that can damage different essential proteins, lipids, and chloroplast membranes under heat stress as compared to the H_sus_ genotype. Similarly, a previous study on ryegrass showed the downregulation of the photosynthetic NDH subunit of the subcomplex under salt stress had an inhibitory effect on the functioning of photosystem II and reduced net photosynthesis [78] matched with the results of the H_sus_ genotype in the current study. The third important protein was (S)-2-hydroxy-acid oxidase (HAOs). HAOs contribute to ROS detoxification by participating in photorespiration and preventing ROS accumulation under heat-stress conditions. Its role in the glyoxylate metabolism pathway contributes to the balance of cellular redox status, ensuring the plant’s ability to cope with stress [79]. According to the previous findings, HAOs present in the peroxisome contribute to the detoxification of hydrogen peroxide under heat stress conditions [80] and the abundance of this enzyme in the H_tol_ genotype helps in the detoxification of reactive oxygen species and regulates the photorespiration process of the plant.

Phosphoribulokinase (PRK) is associated with the Calvin cycle and is directly related to the photosynthetic capacity of plants, the production of HSPs, and many stress signaling pathways. Surprisingly, in our results, PRK was downregulated in the H_sus_genotype, and its lower abundance led to the reduction in net photosynthetic rate (*Pn*) and chlorophyll contents, which makes the cotton plant more sensitive to heat stress. Contrary to previous findings, a rice heat-tolerant strain showed an increase in the expression of PRK, which increases the biosynthesis of thiamine and heat shock proteins (Cpn60, HSP90, and HSP70) under heat stress [81].

All the proteins related to the photosynthetic metabolic pathway inhibited by heat stress and upregulated proteins in the H_tol_ genotype might have protected the plant chloroplast and maintained the photosynthetic activity from ROS by producing heat shock and other antioxidant proteins. Our physiological results demonstrated that the H_sus_ genotype’s propensity to tolerate heat stress conditions with lower photosynthetic rates and chlorophyll levels was diminished by the downregulation of PRK.

### Stress-responsive proteins in H_tol_ genotype

Stress-responsive proteins play a critical role in mediating the cellular response to various biotic and abiotic stresses, including chaperones, antioxidant enzymes, HSPs, and signal transducers, among others. Previous studies reported that, although stress-responsive proteins also participate in other mechanisms like cellular metabolism, gene expression, and stabilization of cellular structures, their main role is to regulate cellular homeostasis by producing ROS-scavenging enzymes under stress conditions [82].

In our study, H_tol_ genotype exhibits a significant upregulation of stress-responsive proteins, approximately double that of the H_sus_ genotype (4.7% compared to 2.2%) under heat stress (S5 and S6 Tables). This higher upregulation of stress-responsive proteins in the H_tol_genotype indicates enhanced resilience because it ensures better protein folding, antioxidant defense, and metabolic stability under heat stress. The upregulation of stress-responsive proteins encompasses various important proteins, including peroxidases, superoxide dismutase, glutathione reductase, protein disulfide isomerase, proteases, NADH dehydrogenases, and MLP-like proteins, among others. Findings from earlier studies that support our proteomic analysis of the H_tol_genotype showed that under heat stress, the activities of important enzymes such as glutathione reductase, ascorbate peroxidase, catalase, superoxide dismutase, and peroxidase increased in heat-tolerant genotypes across all developmental stages, in contrast to susceptible genotypes [12]. One of the interesting findings is the upregulation of an important protein, BRISC, and the BRCA1-A complex in the H_tol_genotype and the downregulation in the H_sus_genotype (Table 1). BRISC and BRCA1-A complex proteins play a significant role in the damage repair of the DNA double strand, ubiquitin signaling regulation, immune signaling, and the metabolism of nucleotides and amino acids [12]. Their role in degrading misfolded proteins and maintaining the concentration of active proteins highlights their significance for cellular homeostasis under stress. BRISC and BRCA1-A complex proteins possess deubiquitinase activity, degrade numerous misfolded and unfolded HSPs, and thereby maintain the normal concentration of active proteins. Furthermore, the upregulation of this protein in the H_tol_ genotype suggested its potential role in the functioning of ROS and HSPs in response to heat stress to stabilize cellular homeostasis. Previous studies on BRISC and BRCA1-A complex proteins revealed that the absence of protein in susceptible genotypes produced higher damage to DNA as compared to wild plants under irradiation stress [83] and similarly in our study, that reduced abundance of this protein in the H_sus_ genotype increased the risk of accumulation of misfolded and unfolded proteins, which leads to the susceptibility.

Many other heat shock proteins, like Hsp70, Hsp90, Hsp80, chaperone proteins, heat shock cognate proteins, luminal binding proteins, and late embryogenesis proteins, were also identified as associated with responses to abiotic and endogenous stimuli. We identified downregulation of three Hsp70 proteins, three Hsp90 isoforms, and Hsps70-Hsps90 organizing proteins in the H_sus_ genotype, which are a significant class of molecular chaperones that are connected to protein folding and facilitate the refolding of denatured proteins, preventing protein aggregation, and stabilizing cell membrane integrity under stress (S6 Table). It was previously reported that Hsp70 supports native protein refolding in both normal and stressful situations by inhibiting protein aggregation [55]. Compared with our physiological, biochemical, and proteomic findings, wild-type tobacco plants overexpressing Hsp70 had reduced levels of reactive oxygen species (ROS), higher net photosynthetic activity, as well as increased proline contents as observed in our study in H_tol_ genotype [84].

Similarly, Hsp90 isoforms and Hsps70-Hsps90 organizing proteins interact with signaling proteins, including transcription factors and kinases. Hsp90 contributes to proteostasis, the cellular balance of protein synthesis, folding, and degradation, which is essential for the overall stress tolerance and adaptation of cotton plants to high-temperature stress. Previous studies reported that Hsp90 only contributes to stress signaling pathways and interacts with heat shock transcription factors (HSFs), which are the key regulators of heat shock responses under heat stress [85]. The downregulation of Hsps in the leaves of H_sus_ plants makes them vulnerable to heat stress due to the aggregation of misfolded and unfolded proteins. This vulnerability leads to impaired protein folding, increased oxidative damage, and loss of membrane integrity, which compromises the plant’s ability to survive heat stress. The luminal binding protein-5 (BiP) was an essential protein that was upregulated in H_tol_ plants and downregulated in H_sus_ plants and played a significant role in the binding of unfolded and misfolded proteins under abiotic stress conditions (Table 1) Previous studies confer the role of BiP as a sensor for disturbances in protein folding and send this misfolded protein out of the endoplasmic reticulum for degradation [86]. In light of previous studies on tobacco and soybean, BiP overexpression in tolerant plants decreased leaf senescence during drought stress [87] and we can correlate our findings that the BiP abundance in the leaves of the tolerant cotton genotype stabilizes the osmotic balance and protein synthesis.

Another interesting finding in our study was the upregulation of MLP-423 protein in the H_tol_ genotype under heat stress, which enhanced the adaptation of plants via abscisic acid-activated signaling pathways (Table 1). MLP-423, like other Hsp70, Hsp90, and other heat shock cognate proteins, participated in protein folding, protein aggregation, regulation of antioxidant enzymes, and other stress-responsive proteins to protect the cotton plant from the detrimental effects of heat stress.From different studies, it was noted that MLP-423 also participated in plant growth and development, including biotic and abiotic stress tolerance [88]. In addition to these roles, MLP-423 contributes to cell wall strengthening and osmotic regulation, which are essential for maintaining structural integrity and water balance under stress [89]. It was reported previously that overexpression of MLP-423 in brown spot-resistant tobacco cultivators may strengthen their resistance to disease by controlling cell wall construction and improving energy metabolism [90].

MLP-423 protein via endogenous ABA content helps to maintain water balance and improve heat tolerance by controlling stomatal closure [91]. These findings strongly supported the morphological and physiological changes in our study, where plant growth and development, transpiration rate (*E*), stomatal conductance (C), and net photosynthetic rate (*Pn*) were maintained by the upregulation of MLA-423 protein in the H_tol_ genotype under heat stress.

All the stress-responsive proteins were mostly the molecular chaperones that were involved in the refolding of unfolded or misfolded proteins, preventing protein aggregation, regulating ROS, and other antioxidant enzymes that alleviated heat stress conditions in tolerant cotton genotypes as compared to H_sus_ genotypes in order to maintain membrane integrity, cellular redox balance, and boost the tolerance mechanism.

### Downregulation of oxidoreductase enzymes affecting the tolerance of H_sus_ genotype under heat stress

Our current research on cotton leaves revealed differential expression of key oxidoreductase proteins, including peroxidases, L-ascorbate oxidase, NADPH dehydrogenase, quinone oxidoreductase, 2-hydroxy acid oxidases, glutathione reductase, and glutathione peroxidase, which are crucial for managing oxidative stress. In the heat-susceptible (H_sus_) genotype, 5.1% of these proteins were downregulated, compared to only 3.7% in the heat-tolerant (H_tol_) genotype (S5 and S6 Tables). This disparity affected essential metabolic pathways in H_sus_, including carbohydrate metabolism, the TCA cycle, photosynthesis, and the production of ROS scavenging enzymes. Such reductions in protein expressions likely compromise the Hsus genotype’s ability to withstand heat stress, similar to findings of previous studies where downregulated antioxidant pathways correlate with decreased stress tolerance. Among the proteomics studies, secoisolariciresinol dehydrogenase (SIRD) showed contrary regulation patterns in the two genotypes; it was upregulated in H_tol_ but downregulated in H_sus_ (Table 1). SIRD, a NAD^+^dependent enzyme, is central to lignan biosynthesis that strengthens the plant cell wall[92]. Previous studies have shown that SIRD upregulation in *Isatisindigotica* roots enhances antiviral lignin production, which reinforces plant defences against various stresses. In our cotton study, SIRD upregulation in H_tol_ likely strengthens lignin production, helping to eliminate ROS and potentially regulating ABA signaling, thereby increasing tolerance to both abiotic and biotic stress [93]. In contrast, reduced SIRD activity in H_sus_ may impair these defenses, limiting lignin synthesis and contributing to lower stress tolerance [94].

Another key finding was the downregulation of 1-aminocyclopropane-1-carboxylate oxidase (ACCO) in H_sus_ under heat stress, while its expression remained steady in H_tol_ (Table 1). ACCO, an enzyme involved in ethylene biosynthesis, is critical for controlling ethylene levels, which are tightly regulated due to their influence on various physiological processes, including photosynthesis, proline and glycine betaine metabolism, and antioxidant defense [95–98]. Previous research shows that balanced ethylene levels, modulated by ACCO, help manage growth and stress responses across plant species [99] Because ethylene assists as a signaling molecule, it is soluble in lipids and diffuses more easily across membranes [100]. In our study, downregulated ACCO in H_sus_ may lead to uncontrolled ethylene production, causing premature leaf senescence, fruit drop, and other developmental challenges under heat stress. In H_tol_, normal ACCO levels appeared to maintain balanced ethylene production, likely contributing to better growth and development during stress [101]That was confirmed by the physiological studies of both genotypes, where the H_tol_ genotype maintained the height, nodes, and the number of fruit-bearing at the first and second positions of branches, as shown in Fig 1. Additionally, controlled ACCO expression in H_tol_ may have facilitated higher proline metabolism, as observed in our results shown in Fig 4B, enhancing cellular dehydration recovery and osmotic adjustment [102].

Our findings thus reinforce the previous studies that underscore the importance of SIRD and ACCO regulation in heat stress adaptation. Upregulation of these proteins in the H_tol_ genotype suggests a coordinated mechanism that balances ROS detoxification and ethylene regulation, crucial for both vegetative and reproductive growth under heat stress. Together, the coordinated regulation of SIRD and ACCO ensures maintaining oxidative balance and developmental stability, providing insight into how strategic protein regulation can enhance plant resilience.

### Transporter activity enhances the tolerance of H_tol_genotype under heat stress

The current finding of our study emphasizes the role of transporter proteins in supporting heat stress tolerance in cotton, particularly having functions such as ion balance, metabolite transport, water regulation, and antioxidant movement. In the H_tol_ genotype, 11 transporter proteins were upregulated, including 4 primary active transporters, 4 channels and pores, and 3 electrochemical potential-driven transporters, as shown in Fig 11 (S5 Table).

Our proteomic analysis identified NADH ubiquinone oxidoreductase (Complex I) as a critical protein that is upregulated in the H_tol_ genotype, supporting ATP production and electron transfer through oxidative phosphorylation under heat stress (Table 1). NADH ubiquinone oxidoreductase upregulation is essential for managing cellular energy requirements and redox homeostasis by facilitating electron transport and reducing ROS production. Consistent with previous studies on maize, Complex I is known to be susceptible to oxidative damage under stress conditions like high salinity, where antioxidants and heat-shock proteins (Hsps) protect Complex I from heat stress [103]. In the current study, upregulation of this complex I protein in the H_tol_genotype ensures its role in cellular stress responses by supporting ATP production and controlling ROS levels.

Two aquaporin plasma intrinsic proteins, PIP2-2 and TIP1-3, were found in the H_tol_genotype (Table 1). The increased expression of ABA-responsive proteins in the H_tol_ genotype suggests better water management because of optimized stomatal control, which helps maintain photosynthetic activity under heat stress. Aquaporins are integral membrane proteins that facilitate the transport of water and other neutral molecules across the membrane and participate in maintaining the water and nutrient uptake, transpiration rate, osmotic regulation, and turgor pressure under stress. According to different studies, AQPs play a significant role in responding to salt and drought stresses in different plants [104]. According to [105], osmotic and salt tolerance were mostly controlled by PIP2-2 in upland cotton. The rise in the concentration of PIP2-2 in the plasma membrane under B excess in broccoli plants increases water uptake under saline stress and increases B uptake in deficiencies [106]. So, like previous studies, the upregulation of these aquaporin proteins in the current study helps to maintain the transpiration rate, stomatal conductance, and net photosynthetic rate in H_tol_ plants under heat stress as seen in Fig 3B–3D.

Our study found that ERD6-6, a key sugar transporter in the electrochemical potential-driven transporter family, is upregulated in the H_tol_ genotype, playing a crucial role in managing osmotic balance by sugar transport (Table 1). ERD6-6 helps to maintain cell turgor and water balance, similar to aquaporins. Previous studies show that ABA suppresses ERD6 in leaves under high salinity stress while upregulating its concentration under drought and low temperature [107]. In Arabidopsis, ERD6 functions to regulate cold tolerance by coordinately modulating sugar and sterol biosynthesis, and both sugars (glucose, sucrose) and sterols work together to mitigate the osmotic stress and protect the membranes more powerfully in citrus [108]. ERD6 helps move glucose out of the storage space (vacuole) in plants when they need to use their stored sugars [109]. Similar to previous research, the upregulation of ERD6-6 in H_tol_ genotype supports osmotic regulation and nutrient uptake, boosting ATP production and cellular redox balance, which ultimately enhances the heat tolerance of plants.

## Conclusion

Rising global temperatures necessitate the understanding of plant response to develop heat-resilient cotton genotypes. This study comprehensively explored the impact of temperature on heat-tolerant and heat-susceptible genotypes. Proteomic analysis revealed that some proteins, like beta-glucosidase, Mg-protoporphyrin IX chelatase, and NDH subunit of subcomplex B 1, were either downregulated or absent in susceptible plants that showed reduced growth, fruit production, and net photosynthetic rates. Some stress-responsive proteins, like BRISC and BRCA1-A complex, luminal-binding protein 5, and MLP-like protein 423, were only upregulated in tolerant plants, linked to immune response, protein metabolism, and cell wall integrity. The upregulation of important proteins, i.e., SIRD and ACCO, is mainly involved in oxidative stress response via controlled ethylene and ABA production in tolerant genotypes to protect the plant from ROS and maintain cellular homeostasis under heat stress. Some transporter proteins, like aquaporin PIP2-2, aquaporin TIP1-3, and ERD6-6, are present only in tolerant genotypes and help in regulating the osmotic balance and nutrients through the ABA signaling pathways and generate energy under stress conditions.

To the best of our knowledge, this is the first study that integrated knowledge from morphophysiological, biochemical, and proteomics to study cotton leaf responses to heat stress. These findings deepen our understanding of protein modifications under heat stress, paving the way for future research to determine whether these proteins help to enhance heat tolerance and aid in the development of more robust cotton varieties.

## Supporting information

S1 Table Proteins identified in the leaves of Htol and Hsus genotypes through LC-MS/MS analysis. (XLSX)

S2 Table Significantly differentially expressed proteins identified in the leaves of Htol genotype. (XLSX)

S3 Table Significantly differentially expressed proteins identified in the leaves of Hsus genotype. (XLSX)

S4 Table Gene ontology functional classification of the differentially expressed downregulated proteins identified in the leaves of Htol genotype. (XLSX)

S5 Table Gene ontology functional classification of the differentially expressed upregulated proteins identified in the leaves of Htol genotype. (XLSX)

S6 Table Gene ontology functional classification of the differentially expressed downregulated proteins identified in the leaves of Hsus genotype. (XLSX)

S7 Table Gene ontology functional classification of the differentially expressed upregulated proteins identified in the leaves of Hsus genotype. (XLSX)
